# SARS-CoV-2 ferritin nanoparticle vaccine induces robust innate immune activity driving polyfunctional spike-specific T cell responses

**DOI:** 10.1038/s41541-021-00414-4

**Published:** 2021-12-13

**Authors:** Joshua M. Carmen, Shikha Shrivastava, Zhongyan Lu, Alexander Anderson, Elaine B. Morrison, Rajeshwer S. Sankhala, Wei-Hung Chen, William C. Chang, Jessica S. Bolton, Gary R. Matyas, Nelson L. Michael, M. Gordon Joyce, Kayvon Modjarrad, Jeffrey R. Currier, Elke Bergmann-Leitner, Allison M. W. Malloy, Mangala Rao

**Affiliations:** 1 Laboratory of Adjuvant and Antigen Research, US Military HIV Research Program, Walter Reed Army Institute of Research, Silver Spring, MD USA; 2 US Military HIV Research Program, Henry M. Jackson Foundation for the Advancement of Military Medicine, Bethesda, MD USA; 3 Department of Pediatrics, F. Edward Hebert School of Medicine, Uniformed Services University of the Health Sciences, Bethesda, MD USA; 4 Oak Ridge Institute of Science and Education, Oak Ridge, TN USA; 5 Emerging Infectious Diseases Branch, Center of Infectious Disease Research, Walter Reed Army Institute of Research, Silver Spring, MD USA; 6 Emerging Infectious Diseases Branch, Henry M. Jackson Foundation for the Advancement of Military Medicine, Bethesda, MD USA; 7 Malaria Biologics Branch, Walter Reed Army Institute of Research, Silver Spring, MD USA; 8 Center for Infectious Disease Research, Walter Reed Army Institute of Research, Silver Spring, MD USA; 9 Viral Diseases Branch, Walter Reed Army Institute of Research, Silver Spring, MD USA

**Keywords:** Vaccines, Infectious diseases

## Abstract

The emergence of variants of concern, some with reduced susceptibility to COVID-19 vaccines underscores consideration for the understanding of vaccine design that optimizes induction of effective cellular and humoral immune responses. We assessed a SARS-CoV-2 spike-ferritin nanoparticle (SpFN) immunogen paired with two distinct adjuvants, Alhydrogel^®^ or Army Liposome Formulation containing QS-21 (ALFQ) for unique vaccine evoked immune signatures. Recruitment of highly activated multifaceted antigen-presenting cells to the lymph nodes of SpFN+ALFQ vaccinated mice was associated with an increased frequency of polyfunctional spike-specific memory CD4^+^ T cells and K^b^ spike-(539–546)-specific long-lived memory CD8^+^ T cells with effective cytolytic function and distribution to the lungs. The presence of this epitope in SARS-CoV, suggests that generation of cross-reactive T cells may be induced against other coronavirus strains. Our study reveals that a nanoparticle vaccine, combined with a potent adjuvant that effectively engages innate immune cells, enhances SARS-CoV-2-specific durable adaptive immune T cell responses.

## Introduction

Coronaviruses (CoV) are positive-sense, single-stranded RNA viruses that cause varying disease pathologies ranging from common cold symptoms to acute respiratory distress syndrome, as well as, gastrointestinal symptoms^1–3^. SARS-CoV-2, the causative agent of COVID-19, represents the seventh CoV to be isolated from humans and is the third to cause severe disease^4^. The rapid and unparalleled spread of SARS-CoV-2 into a global pandemic has driven an urgent need for rapidly deployable and scalable vaccine platforms. A myriad of vaccine platforms and approaches have been adopted and developed by government, industry, academic, and nongovernmental organizations. Two messenger RNA-based vaccines and an adenovirus vector-based vaccine have been approved for emergency use in the United States^5^ (https://www.raps.org/news-and-articles/news-articles/2020/3/covid-19-vaccine-tracker). A recombinant nanoparticle vaccine, NVX-CoV2373, that contains the full-length spike glycoprotein and a saponin Matrix-M1 adjuvant, administered as a two-dose regimen to adult participants in a phase 3 trial, conferred 89.7% protection against SARS-CoV-2 infection and showed high efficacy against the B.1.1.7 variant^6^. Messenger RNA-based vaccines^7,8^ and recombinant adenovirus vectored vaccines^9–11^ have also demonstrated potent efficacy and varying degrees of cross-reactivity to variants of concern^12^. The recent emergence of rapidly evolving immunological variants^13–15^ and concern over future variants that might more fully evade vaccine-induced immunity raises the issue of how different arms of the adaptive immune response are elicited by vaccines for optimization for SARS-CoV-2 and future pandemics.

Naturally occurring nanoparticle vaccines such as Yellow Fever 17D vaccine^16^ and human papillomavirus virus-like particle (VLP) vaccines^17^ elicit robust and long-lived immune responses. This is because the multivalent presentation of antigens significantly enhances the immune response and several nanoparticle-based platforms have been utilized for this purpose. Ferritin is one such self-assembling multimeric vaccine platform that has been used to display antigens such as influenza, HIV-1, Epstein-Barr virus, and SARS-CoV-2^18–21^. The latter, induced robust immune responses to SARS-CoV-2 in mouse studies when combined with Quil A and MPL as the adjuvant^21^.

We have recently developed a SARS-CoV-2 sub-unit vaccine based on a ferritin nanoparticle platform^18^ that displays a pre-fusion stabilized spike on its surface^22,23^. The spike protein was modified to generate a stable spike trimer formation on the ferritin molecule^23,24^. The stabilized prefusion-spike protein of the Wuhan-Hu-1 strain of SARS-CoV-2 was genetically linked to form a ferritin-fusion recombinant protein, which naturally forms a Spike Ferritin nanoparticle (SpFN). Ferritin is a naturally occurring, ubiquitous, iron-carrying protein that self-oligomerizes into a 24-unit spherical particle and is currently being evaluated as a vaccine platform for influenza in two phase 1 clinical trials (NCT03186781, NCT03814720) with two further trials in the recruitment phase for Epstein-Barr virus (NCT04645147) and Influenza H10 (NCT04579250).

The immune response to SpFN was evaluated by formulating it with one of the most commonly used adjuvants, Aluminum Hydroxide gel (Alhydrogel^®^) (AH) or with Army Liposome Formulation containing the saponin, QS-21 (ALFQ) based on our prior experience with this adjuvant formulation. ALFQ contains two immunostimulants, synthetic MPLA (3D-PHAD^®^) and QS-21^25^, the same two immunostimulants that are also present in AS01B, the adjuvant in the highly efficacious licensed herpes-zoster vaccine, Shingrix^TM ^^26^. However, the liposomes in ALFQ fundamentally differ from those in AS01B, both in the phospholipid type and cholesterol content in addition to differences in the amount of 3D- PHAD^®^ and QS-21. Previously, it has been shown in several different models that ALFQ generates well-balanced T_H_1/T_H_2 immunity and protective efficacy^27–29^. Another vaccine NVX-CoV2373 also contains a saponin-based adjuvant. It is not a specific saponin fraction, but a mixture of saponins from the bark of the tree *Quillaja saponaria Molina* formulated with cholesterol and phospholipid to form 40 nm nanoparticles (Matrix-M1). This formulation does not contain the additional immunostimulant 3D-PHAD^®^. In rodent and nonhuman primate challenge models, NVX-CoV2373 induced high titers of neutralizing antibodies and polyfunctional CD4^+^ and CD8^+^ T cell responses with a T_H_1 dominant phenotype^30^.

Immunogenicity and challenge studies in mice^23^, hamsters^31^, and nonhuman primates^32^ following vaccination with SpFN+ALFQ showed robust antibody responses to spike protein and protection against challenge with SARS-related Coronavirus 2, Isolate USA-WA1/2020 and B.1.1.7 and B.1.351 virus variants. A phase 1 clinical trial (ClinicalTrials.gov Identifier: NCT04784767) with SpFN+ALFQ is currently ongoing.

In this study, we show the complex signature of antigen-presenting cell (APC) engagement within the lymph nodes draining the vaccination site (dLN) and its impact on a SARS-CoV-2 vaccine-induced adaptive immunity. We demonstrate that SpFN formulated with ALFQ (SpFN + ALFQ) compared to SpFN formulated with AH (SpFN + AH) significantly increased the number and activation of classical and nonclassical APCs. Intriguingly, this was associated with a potent T_H_1 cellular response and highly functional spike-specific memory T cells. The APC response to SpFN+ALFQ was characterized by conventional type 1 and type 2 dendritic cells (cDC1 and cDC2) with upregulated costimulatory molecules. The effective APC response induced by SpFN+ALFQ correlated with differentiation of spike-specific CD4^+^ T cells expressing markers of T_H_1 phenotype. Strikingly, vaccination with SpFN+ALFQ resulted in spike-specific CD8^+^ T cells that established a memory pool. We identified eleven SARS-CoV-2 T cell epitopes in C57BL/6 mice vaccinated with SpFN+ALFQ that mapped to the spike protein corroborated by the findings of others^33^. Using an MHC class I tetramer, we identified murine K^b^ restricted SARS-CoV-2-specific memory CD8^+^ T cells recognizing an eight amino acid sequence (amino acids 539–546; VNFNFNGL) of the SARS-CoV-2 spike protein that is conserved in the SARS-CoV spike protein. The K^b^ spike _(539–546)_-specific memory CD8^+^ T cells generated by vaccination with SpFN+ALFQ exhibited significantly more cytotoxic activity compared to those identified in the SpFN+AH vaccinated mice. CD69^+^ CD103^+^ K^b^ spike _(539–546)_-specific CD8^+^ T cells could be identified in perfused lung tissue of SpFN+ALFQ vaccinated mice at 6 weeks and 23 weeks post-vaccination. Together these findings demonstrate that this vaccine platform for SARS-CoV-2 leverages the innate immune response to induce potent memory-specific antiviral T cells.

## Results

### Spike ferritin nanoparticle (SpFN) adjuvanted with ALFQ (SpFN + ALFQ) induced a more robust and sustained APC response compared to formulation with aluminum hydroxide (SpFN + AH)

**Fig. 1:**
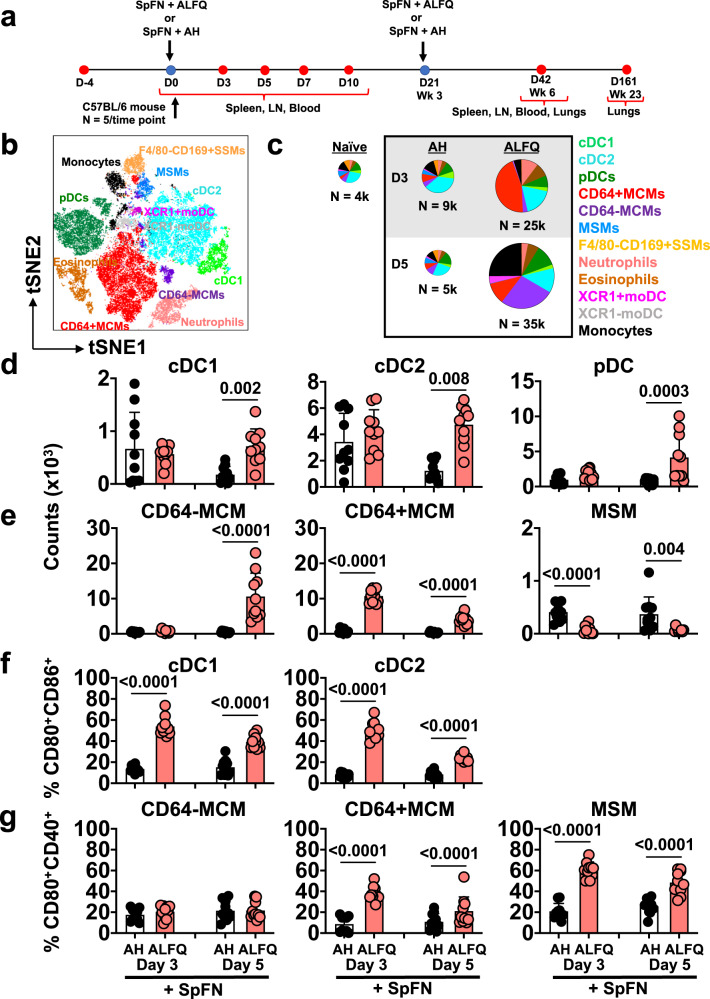
Enhanced APC recruitment in response to a SARS-CoV-2 vaccine (SpFN) adjuvanted with ALFQ (SpFN + ALFQ) compared to AH (SpFN + AH). **a** Vaccination and sample collection schedule. C57BL/6 mice received a prime (week 0)-boost (week 21) vaccination regimen with SpFN formulated with either AH (SpFN + AH) or ALFQ (SpFN + ALFQ). At days 0, 3, 5, 7, and 10, spleen, lymph nodes draining the left quadriceps (dLN), and blood were collected. At week 6 (day 42) post-first vaccination, spleen, mediastinal, and perfused lungs were collected. At week 23 (day 161), perfused lungs were collected. **b** The t-SNE display of the APC subsets identified by the gating strategy is shown in Supplementary Fig. 1A**. c** Pie charts comparing the number of recruited APCs measured in the dLNs at days 3 and 5 in response to the two vaccine formulations (*n* = 5/group/time point) or from naïve mice (*n* = 4). Twelve APC subsets were identified. Each slice indicates an APC subset; N indicates the average of the total number of APCs quantified in the dLNs. **d**, **e** The number of **d** conventional (cDC1, cDC2) and plasmacytoid DCs (pDCs) and **e** Medullary cord and medullary sinus macrophage (MCM and MSM) subsets in both vaccine groups at indicated time points. **f** Activation of cDCs indicated by CD80 and CD86 and **g** macrophage subset activation indicated by CD80 and CD40 in both vaccine groups at indicated time points. Bars indicate mean + s.d. SSM subcapsular sinus macrophages, MoDC monocyte-derived DC. Experiments were repeated twice and differences between the two groups were analyzed by using a nonparametric Mann–Whitney *U*-test with *p* ≤ 0.05 considered as statistically significant.

Adjuvants influence the engagement and activation of innate immune cells to facilitate vaccine antigen presentation^34,35^. To define the ability of SpFN + ALFQ to engage the innate immune response, we compared our vaccine formulations with SpFN + AH. Mice were vaccinated at the specified time points and innate immune cells were analyzed from the dLNs (inguinal and popliteal lymph nodes) at days 3 and 5 post-vaccination (Fig. 1a). A multiparameter spectral flow cytometry panel (Supplementary Table 1) was used to discriminate APC subsets. T-distributed Stochastic Neighbor Embedding (tSNE) was used to visualize the high-dimensional datasets (Fig. 1b). The flow cytometric gating strategy is shown in Supplementary Fig. 1a. Significant differences in the composition of APCs present in the dLNs were seen in mice vaccinated with SpFN + ALFQ compared to SpFN + AH (Fig. 1c and Supplementary Fig. 1b, c). The overall number of APCs present in the dLNs increased 3–7-fold in SpFN+ALFQ compared to SpFN + AH vaccinated mice, as shown by the pie graphs in Fig. 1c. Interestingly, the number and diversity of APCs began to contract 5 days post-vaccination in the SpFN + AH vaccinated mice to levels exhibited in naïve mice. In contrast, mice vaccinated with SpFN + ALFQ exhibited continued recruitment or expansion of APCs in the dLNs at this time point, demonstrating a more sustained response to the adjuvant formulation, ALFQ (Fig. 1c).

Furthermore, not only were the numbers of APCs increased after SpFN + ALFQ vaccination at days 3 and 5, but classical APCs, such as migratory DCs and LN resident APCs, predominated (Fig. 1d). Migratory DCs, which include cDC1 and cDC2s, migrate from tissue sites to dLNs to present antigen and support T cell activation and differentiation and were increased in number in the SpFN + ALFQ vaccinated mice (Fig. 1d). In addition, plasmacytoid DCs (pDCs), that provide type I interferon for DC and T cell activation, were also increased in the dLNs after the priming vaccination with SpFN + ALFQ compared to SpFN + AH (Fig. 1d).

Lymph node resident macrophages play a role in the acquisition of antigens through pinocytosis and phagocytosis and participate in immune regulation. Medullary sinus macrophages (MSM) defined as CD169^+^CD11b^+^F4/80^+^MHCII^int^ CD11c^lo^ and medullary cord macrophages (MCM) defined as CD169^−^CD11b^+^F4/80^+^MHCII^int^ CD11c^lo^CD64^+/−^^36^ were the most significantly divergent (*p* < 0.0001) between the two vaccinated groups; higher numbers of MCM were measured in the SpFN + ALFQ vaccinated mice, whereas MSM numbers were significantly higher in the SpFN + AH vaccinated mice. Subcapsular sinus macrophages (SSM), which have been shown to uptake QS-21^37^, were identified as CD11b^+^CD169^+^F4/80^−^ cells (Supplementary Fig. 1a). CD64^−^ and CD64^+^MCM were infrequent in numbers on both days 3 and 5 in the SpFN + AH vaccinated mice, while CD64^+^ MCM were present in higher numbers on both days 3 and 5 post-vaccination with SpFN + ALFQ, and CD64^−^MCM increased by 10,000 cells on day 5 post-SpFN + ALFQ vaccination. In contrast, the number of MSM and SSM was decreased in the dLNs of SpFN + AFLQ vaccinated mice (Fig. 1e and Supplementary Fig. 1d).

Commensurate with increased numbers of classical APCs, the functional activation of these cells was also enhanced by SpFN + ALFQ. In addition to antigen presentation, costimulatory molecules, such as CD80 and CD86, are expressed by activated DCs and engage T cells, inducing activation in response to ligation with their peptide-MHC complex^38,39^. The percentage of cDC1 and cDC2 expressing CD80 and CD86 three days post-vaccination was three times higher in the SpFN + ALFQ group compared to SpFN+AH (Fig. 1f and Supplementary Fig. 1b). We also measured the activation of macrophage subsets. While the expression of CD80 and CD40 were similarly low in CD64^−^MCMs, the CD64^+^MCMs from SpFN + ALFQ vaccinated mice significantly upregulated CD80 and CD40 by threefold at day 3, and twofold at day 5 post-priming vaccination compared to the SpFN + AH group (Fig. 1g). Although the MSM numbers were higher with AH as the adjuvant, the expression of costimulatory molecules, CD80 and CD40, was increased in the SpFN + ALFQ vaccinated mice suggesting a higher activation status (Fig. 1g).

Neutrophils, eosinophils, and monocytes have been shown to support and provide antigen for pathogen- and vaccine-specific T and B cell differentiation in humans and mice^40–44^. Increased numbers of eosinophils, neutrophils, and monocytes were observed in the dLNs of SpFN + ALFQ vaccinated mice at days 3 and 5 post-vaccination (Supplementary Fig. 1e**)**. In addition, a notable proportion of CD11b^+^MHCII^+^ monocyte-like cells were CD11c intermediate, indicative of monocyte-derived DCs (MoDCs), and were further differentiated by expression of the chemokine receptor XCR1 (Fig. 1b and Supplementary Fig. 1a). The XCR1 + MoDCs were distinct from cDC1, as XCR1 + MoDCs were MHC-II low, CD11c low, and CD103 negative, and displayed two separate populations by t-SNE. Few XCR1^+^MoDCs were detected in the dLNs of either vaccine group on day 3 post-priming vaccination. However, by day 5 post-vaccination this MoDC subset had significantly increased in the SpFN + ALFQ group suggesting a potential role in vaccine-specific T cell differentiation (Supplementary Fig. 1e).

### Effective APC induction by vaccination with SpFN + ALFQ was associated with increased SARS-CoV-2 spike-specific T cells in the dLNs

**Fig. 2:**
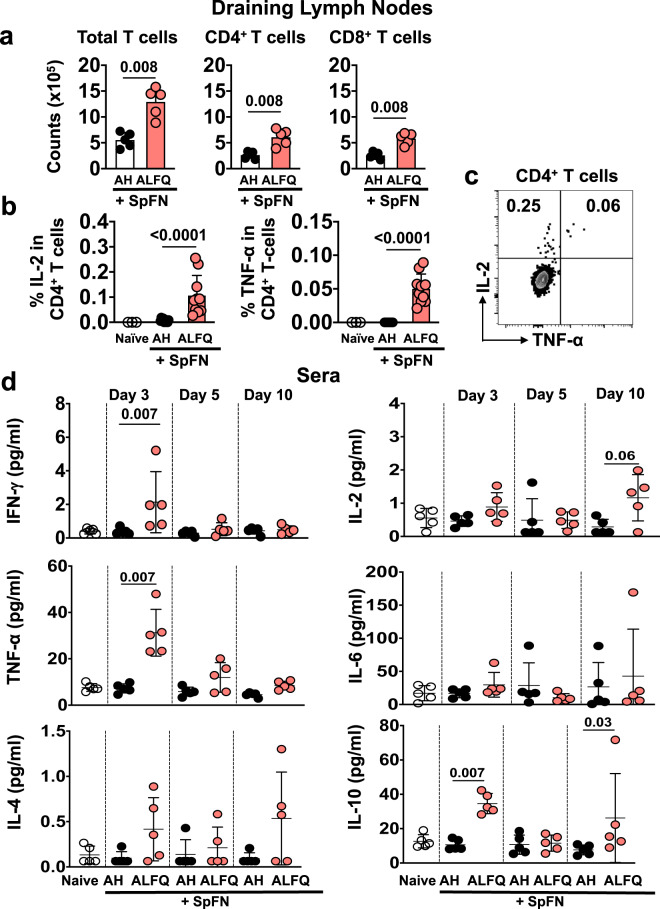
T cell priming and cytokine profile in the sera in response to SpFN vaccine. **a** Magnitude of the T cell response in the dLNs at day 7 post-priming vaccination (*n* = 5 mice/group/time point). **b** Percentage of IL-2 expressing (left panel) and TNF-α expressing (right panel) SARS-CoV-2 spike-specific CD4^+^ T cells in the dLNs 10 days post-vaccination, following stimulation with spike S1 peptide pool. Bars indicate mean + s.d. **c** IL-2 and TNF-α are co-expressed in the SARS-CoV-2 spike-specific CD4^+^ T cells, indicating mainly a T_H_ 1 type response. **d** Cytokine profile in the sera of vaccinated mice as determined by multiplex ECLIA using Meso Scale Discovery (MSD) platform on days 3, 5, and 10 post-vaccination. Dots represent data from individual mice, horizontal lines represent mean, and error bars represent s.d. Experiments were repeated twice and differences between the two groups were analyzed by using a nonparametric Mann–Whitney *U*-test with *p* ≤ 0.05 considered as statistically significant.

We analyzed the recruitment of total and spike-specific T cells to the dLNs and their phenotypic and cytokine expression on days 7 and 10, respectively. At day 7 post-priming vaccination, the total T cells, as well as the CD4^+^ and CD8^+^ T cell subsets, in the dLNs of SpFN+ALFQ vaccinated mice were significantly higher than those in the SpFN + AH vaccinated mice (Fig. 2a). T cell memory differentiation potential was evaluated by the expression of CD62L and CD44 (Supplementary Fig. 2a). In addition to the numeric differences of the T cell subsets, SpFN + ALFQ vaccination resulted in proportionally more effector memory-like (CD62L^−^CD44^+^) CD4^+^ and CD8^+^ T cells in the dLN. In contrast, SpFN + AH vaccination increased the frequency of CD44^−^ T cells (CD62L^+^CD44^−^) in the dLNs, suggesting reduced recognition of cognate antigen (Supplementary Fig. 2b).

At day 10 post-priming vaccination, SARS-CoV-2 spike-specific T cells were identified by intracellular cytokine staining following stimulation with a spike peptide pool (S1) containing the epitopes in the receptor-binding domain (RBD). Spike-specific CD4^+^ T cells were readily detected by expression of IL-2 and TNF-α upon peptide stimulation in the dLNs of mice vaccinated with SpFN + ALFQ and to a much lower extent (*p* < 0.0001) in those of SpFN + AH vaccinated mice (Fig. 2b). IL-2 and TNF-α were co-expressed in a subset of the CD4^+^ T cells (Fig. 2c), which expressed CD44^+^CD62L^−^CCR7^−^ exhibiting effector memory potential (Supplementary Fig. 2c). The intracellular cytokine responses measured from the spike-specific T cells indicated that the CD4^+^ T cell responses were predominantly directed toward T_H_1 (TNF-α and IL-2) rather than a T_H_2 profile. To further confirm our findings, multiplex cytokine analysis was performed with the sera of mice at days 3, 5, and 10 following the priming vaccination. The T_H_1 cytokines (IFN-γ and TNF-α), as well as IL-10, were significantly higher in the peripheral blood of the SpFN + ALFQ vaccinated mice (Fig. 2d) indicating that the cytokine profile was skewed towards a T_H_1 profile, which was consistent with the measurement of intracellular cytokine expression.

### Early expansion of SARS-CoV-2 spike-specific CD4^+^ and CD8^+^ T cells following priming vaccination with SpFN + ALFQ

**Fig. 3:**
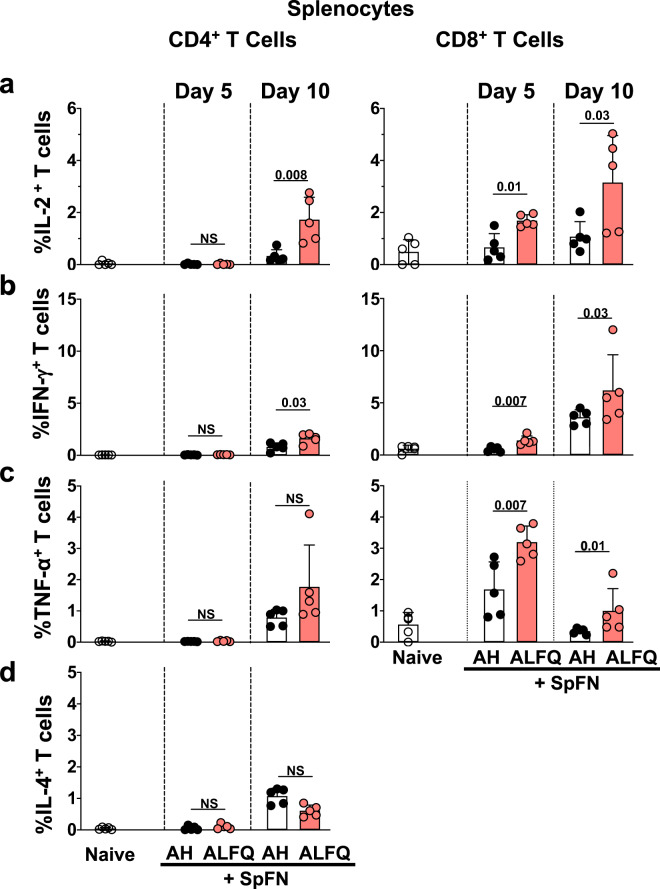
Splenic T cell responses in mice vaccinated with SpFN vaccine. **a**–**c** Flow cytometry-based characterization of mouse splenocytes at days 5 and 10 post-vaccination measuring SARS-CoV-2 spike-specific intracellular cytokine responses in CD4^+^ and CD8^+^ T cells **a** IL-2-, **b** IFN-γ- **c** TNF-α-, and **d** IL-4- secreting T cells. Dots represent data from individual vaccinated or naïve mice (*n* = 5/group). Bars represent mean + s.d. Statistical significance between the groups was determined using a nonparametric Mann–Whitney *U*-test with *p* ≤ 0.05 considered as statistically significant. The gating strategy applied for the evaluation of flow cytometry-acquired data presented in this figure is provided in Supplementary Fig. 4.

Since SpFN + ALFQ triggered more robust APC activation along with significantly higher percentages of functional antigen-specific CD4^+^ T cells in the dLNs, we, therefore, evaluated the phenotype and functional CD4^+^ and CD8^+^ T cell responses in the spleen at days 5 and 10 post-vaccination. Cells were stimulated with SARS-CoV-2 spike-specific peptides from well-characterized S1 and S2 peptide pools, followed by flow cytometric measurement. Although there were no significant differences in the frequency of total T cells, CD4^+^ and CD8^+^ T cells at day 5 and 10 post-vaccination (Supplementary Fig. 3a), we did observe significant differences in the SARS-CoV-2 spike-specific cytokine-expressing CD4^+^ and CD8^+^ T cells. CD4^+^ T cells expressing IL-2 (*p* = 0.008) and IFN-γ (*p* = 0.03) were significantly higher in the ALFQ group compared to the AH group at day 10 (Fig. 3a–c). Although, the CD4^+^ T cells expressing IL-4 were higher on days 5 and 10 post-vaccination for both vaccine groups compared to CD4^+^ T cells from unvaccinated (naïve) mice upon peptide stimulation, there were no significant differences between the AH and the ALFQ groups (Fig. 3d). In addition, CD8^+^ T cells secreting IL-2 (Day 5: *p* = 0.01; Day 10: *p* = 0.03, Supplementary Fig. 3b), IFN-γ (Day 5: *p* = 0.007; Day 10: *p* = 0.03, Supplementary Fig. 3c), and TNF-α (Day 5: *p* = 0.007; Day 10: *p* = 0.01, Supplementary Fig. 3d) were higher in SpFN + ALFQ vaccine group on both days 5 and 10 post-vaccination (Fig. 3a–c). The frequency of cytokine-positive cells was generally higher in the CD8^+^ T cell population than the CD4^+^ T cell population at day 10 compared to day 5 post-vaccination (Fig. 3a–c).

### SARS-CoV-2 spike-specific T cells exhibit increased localization to the lung draining lymph node and a T_H_1 cytokine profile in SpFN + ALFQ vaccinated mice following a prime-boost vaccination

**Fig. 4:**
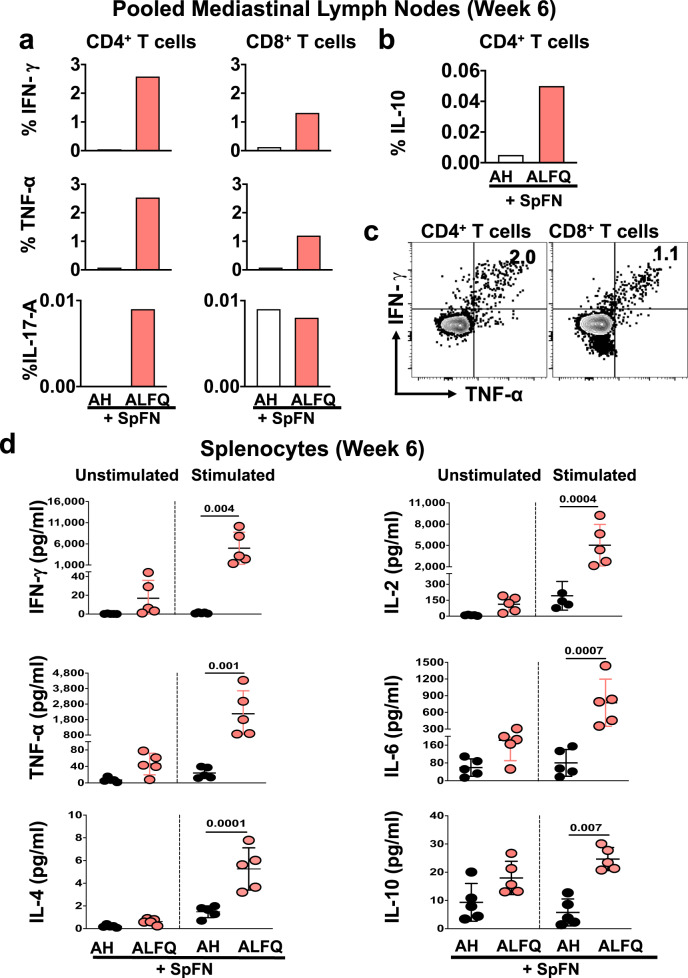
Cytokine responses in the pooled mediastinal lymph nodes and spleens of mice after SpFN prime-boost vaccination. **a**, **b** Percentage of SARS-CoV-2 spike-specific T cells in the mediastinal lymph nodes at week 6 (3 weeks post-prime-boost vaccination). **a** Percentage of CD4^+^ and CD8^+^ T cells expressing IFN-γ, TNF-α, IL-17A, and **b** CD4^+^ T cells expressing IL-10. Each sample is representative of the pooled lymph nodes within the vaccine group. **c** SARS-CoV-2 spike-specific CD4^+^ and CD8^+^ T cells in the mediastinal lymph nodes following prime-boost vaccination with SpFN + ALFQ co-express IFN-γ and TNF-α upon peptide stimulation. **d** Splenocytes were stimulated with SARS-CoV-2 spike-specific peptides and cytokines in the culture supernatants were measured by the ECLIA-based multiplex MSD platform. Dots represent data from individual mice (*n* = 5/group), horizontal lines represent mean and error bars represent s.d. Differences between the two groups were analyzed by using a nonparametric Mann–Whitney *U*-test with *p* ≤ 0.05 considered as statistically significant.

To determine whether an intramuscular vaccination could prime SARS-CoV-2-specific memory T cells to home to tissue sites relevant to potential infection, the T cell response in the mediastinal LNs were measured at week 6 (3 weeks post-boost) post-vaccination. Since the number of cells were limiting, mediastinal LNs were pooled separately for each of the vaccination groups. The gating strategy for the identification of SARS-CoV-2 spike-specific CD4^+^ and CD8^+^ T cells is shown in Supplementary Fig. 2d. Spike-specific CD4^+^ and CD8^+^ T cells expressed IFN-γ, TNF-α, IL-17A (Fig. 4a), and IL-10 (Fig. 4b) in the SpFN + ALFQ vaccine group. The IFN-γ expressing CD4^+^ and CD8^+^ T cells exhibited activation and effector memory markers (CD69^+^ CD44^+^ and CD62L^−^ CD103^−^; Supplementary Fig. 2e, f**)**. Furthermore, IFN-γ and TNF-α were dominant and co-expressed by both spike-specific CD4^+^ and CD8^+^ T cells in the SpFN + ALFQ vaccine group (Fig. 4c). Characterization of the memory phenotype in the IFN-γ^+^ CD4^+^ T cells responsive to the S1 peptide pool showed a predominance of T_EM_ suggesting strong stimulation through the T cell receptor during priming^45^ (Supplemental Fig. 2g).

The profile of pro-inflammatory cytokines was also compared in the spleen at week 6 post-vaccination to track the differentiation of antiviral T cell responses. Splenocytes were stimulated with pooled SARS-CoV-2 spike peptides spanning the S1 and S2 subunits and the cytokine profiles were assessed in the two vaccination groups. IFN-γ, TNF-α, IL-2, and IL-6 levels were significantly increased in SpFN + ALFQ vaccinated mice compared to mice that received SpFN + AH vaccination (Fig. 4d). The IL-4 and IL-10 levels were also elevated in the SpFN + ALFQ vaccinated group, although the magnitude of increase was much lower in comparison to the T_H_1 cytokines (Fig. 4d).

### Prime-boost vaccination with SpFN + ALFQ resulted in a more robust SARS-CoV-2 spike-specific T cell response

**Fig. 5:**
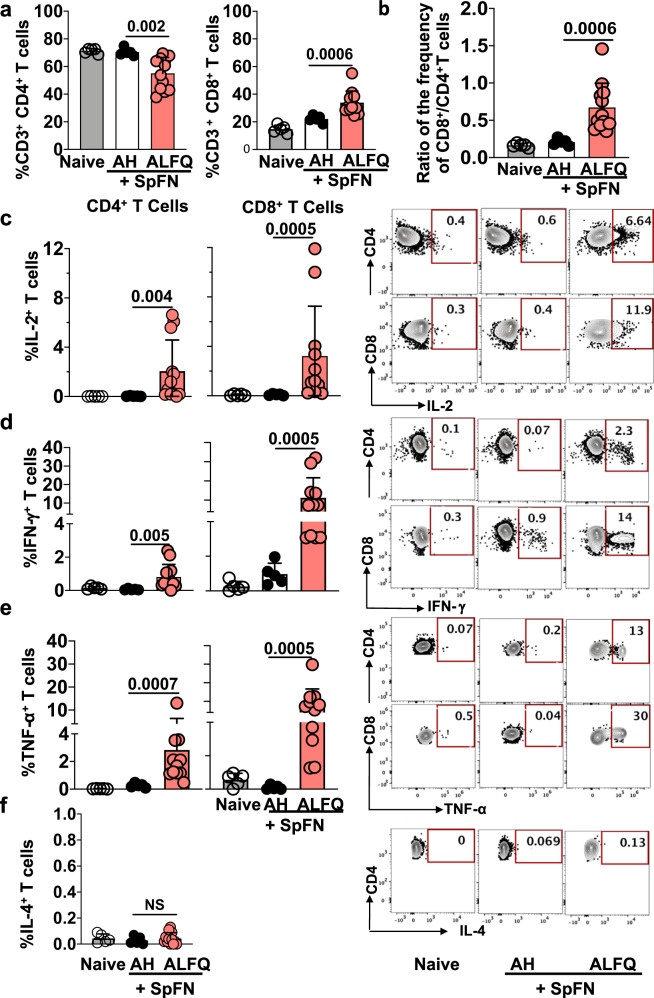
T cell responses in splenocytes of mice in response to SpFN vaccine at week 6 following prime-boost vaccination. **a** Frequency of total CD4^+^ and CD8^+^ T cells. **b** Ratio of the frequency of total CD8^+^/CD4^+^ T cells. Bar graphs and representative flow plots demonstrate cytokine **c** IL-2, **d** IFN-γ, **e** TNF-α secreting CD4^+^ and CD8^+^ T cells, and **f** IL-4 secreting CD4^+^ T cells. Dots represent data from individual mice (naive: *n* = 5; AH: *n* = 5; ALFQ: *n* = 11, from two independent experiments). Bars represent mean + s.d. Statistical significance between the groups was determined using a nonparametric Mann–Whitney *U*-test with *p* ≤ 0.05 considered as statistically significant.

To further assess the durability of T cell responses, we characterized the T cell phenotypes and functional responses in the spleen at week 6 post-vaccination. We observed an expansion of CD8^+^ T cells compared to CD4^+^ T cells as measured by a decrease in the total CD4^+^ (*p* = 0.002) and an increase in the total CD8^+^ T cells (*p* = 0.0006) in SpFN + ALFQ compared to SpFN + AH vaccination group (Fig. 5a, b). To assess the frequency of cytokine-secreting CD4^+^ and CD8^+^ T cells, splenocytes were stimulated ex vivo with SARS-CoV-2 spike peptides spanning the S1 or S2 subunits. The flow gating strategy is shown in Supplementary Fig. 4. The vaccine boost dramatically increased the frequency of SARS-CoV-2 spike S1-specific IL-2 (CD4^+^: *p* = 0.004; CD8^+^: *p* = 0.0005, Fig. 5c), IFN-γ (CD4^+^: *p* = 0.005; CD8^+^: *p* = 0.0005, Fig. 5d), and TNF-α (CD4^+^: *p* = 0.0007; CD8^+^: *p* = 0.0005, Fig. 5e) secreting CD4^+^ and CD8^+^ T cells, while no differences were observed in frequency of IL-4 secreting cells (Fig. 5f). The booster dose of SpFN + ALFQ resulted in a further expansion of cytokine-producing T cells above the priming response at day 5 and/or day 10. This suggested that the two-dose regimen of the SpFN + ALFQ vaccine formulation improved the generation and differentiation of SARS-CoV-2-specific T cell memory responses. Importantly, the S1 peptide pool containing the RBD and NTD portions of the spike protein produced significantly greater cytokine expression compared to the minimal responses generated by the S2 peptide pool.

### T cell responses induced by SpFN + ALFQ are focused on the S1 domain of the SARS-CoV-2 spike protein

**Fig. 6:**
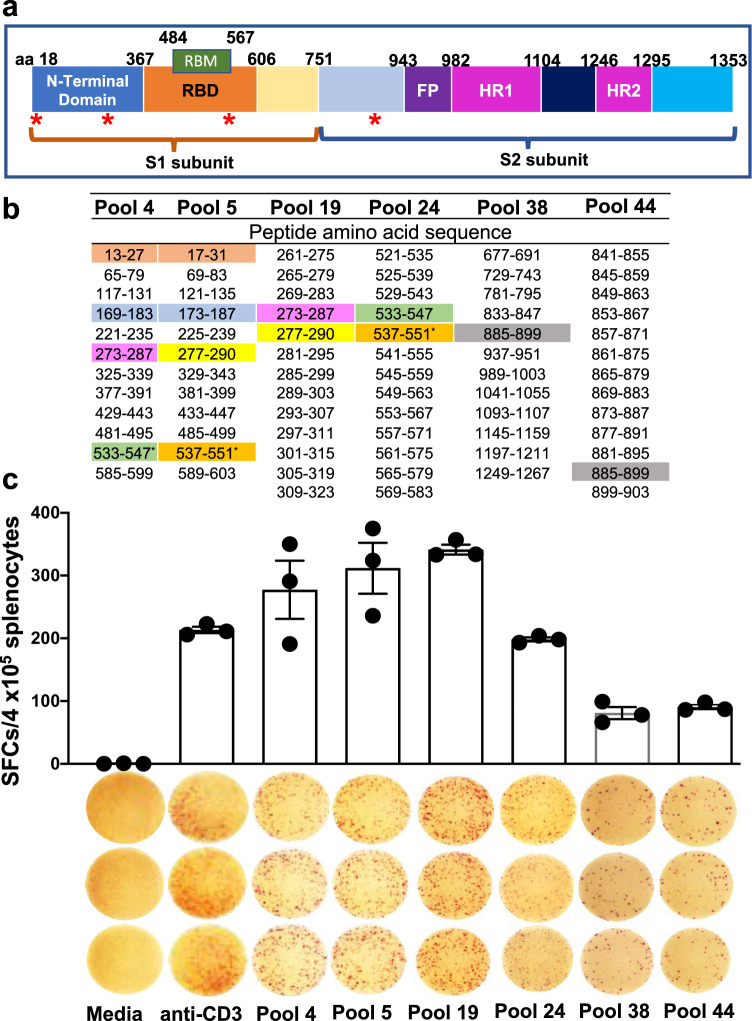
Epitope mapping data in the splenocytes of SpFN vaccinated mice at week 6 following prime-boost vaccination. **a** Schematic representation of the SARS-CoV-2 spike protein showing the different domains with amino acid (aa) numbers. Red asterisk denotes the identified H2K^b^/D^b^-restricted epitopes. **b** Amino acid residues of SARS-CoV-2 glycoprotein peptide pools. Matrix pools (4, 5, and 38) and overlapping peptide pools of 15-mer peptides overlapping by 11 aa (19, 24, and 48) containing 315 peptides across the entire glycoprotein were tested in the ELISpot assay. Reactivity to specific matching sequences in pools are highlighted with different matching colors and represent the predicted epitopes for H2K^b^/D^b^ (iedb.org) or predicted epitopes without ELISpot responses (blue highlight; hypothetical epitope). Amino acid sequences 13–27 (pool 4) and 17–31 (pool 5) have been highlighted in color to indicate confirmed epitopes based on ELISpot reactivity to peptide pool 14. **c** Number of spike-specific IFN-γ spot forming units stimulated with 52 peptide pools from the Epitope Mapping Peptide Set of the spike protein is presented as scatter plots (mean ± s.d./4 × 10^5^ splenocytes). ELISpot images of triplicate wells and the stimulants are shown on the X-axis. T cell responses were considered positive when mean spot count exceeded the mean ± 3 s.d. of the negative control wells. Splenocytes were stimulated in vitro with media (negative control), anti-CD3 (positive control), or SARS-CoV-2 spike peptide pools 4, 5, 19, 24, 38, and 44.

Next, we characterized the peptides of the spike protein targeted by the vaccine-induced T cell response. Our data demonstrated that the cytokine response to SARS-CoV-2 was dominated by IFN-γ, therefore an IFN-γ-specific ELISpot assay was used to screen 52 peptide pools composed of 315 peptides as this methodology requires fewer cells and has a higher sensitivity. Peptides comprised the full length of the spike protein, represented with its major subunits and domains (S1 domain containing the NTD, RBD, S2 domain-containing FP, HR1, and HR2) in Fig. 6a. Peptides were pooled in a matrix scheme to allow the high-throughput screening and identification of potential epitopes (Fig. 6b and Supplementary Fig. 5). Most of the measured reactivity to the peptide pools was focused on the NTD and RBD within the S1 domain of the spike protein with the highest responses in pools 4, 5, and 19 (Fig. 6c). The results revealed one epitope, VNFNFNGL (aa 539–546) that was not predicted by the NetMHCPan 4.1 EL prediction tool (iedb.org) but was previously reported^33^ in mice transfected with human ACE-2 mRNA and subsequently infected with SARS-CoV-2, as well as defined in SARS-CoV^46^. This epitope is conserved between the two viruses. Comparing the NetMHCPan 4.1 EL results with the IFN-γ ELISpot analysis suggest that all epitopes are either H2K^b^ or H2D^b^ restricted and none of them represented CD4^+^ epitopes.

### Induction of K^b^-spike _(539-546)_-specific polyfunctional CD8^+^ T cell responses following booster vaccination with SpFN + ALFQ

**Fig. 7:**
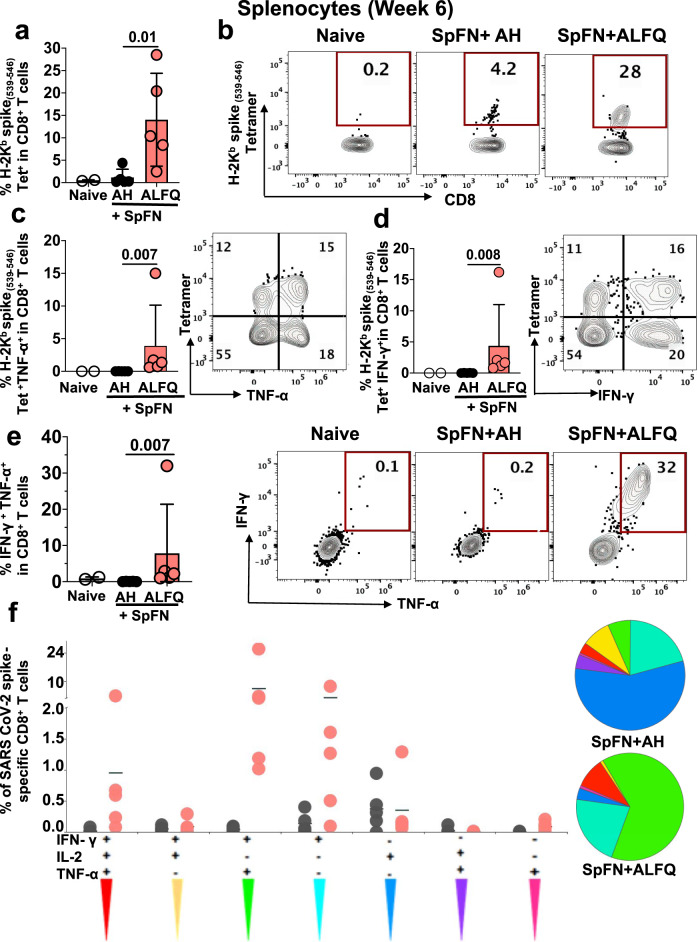
SARS-CoV-2 Kb spike (539–546)-specific MHC class I restricted CD8 + T cells in response to SpFN vaccine following booster vaccination. **a** Frequency of antigen-specific CD8 + T cells in splenocytes of naïve and vaccinated mice as determined by flow cytometry-based SARS-CoV-2 spike (539–546)-specific tetramer staining. **b** Representative flow plots depicting the frequency of Kb spike (539–546)-specific CD8 + T cells at week 6 in all the groups. Bar graphs and representative flow plots demonstrate the significantly higher percentage of spike (539–546)-specific-, **c** TNF-α +, **d** IFN-γ + CD8 + T cells. **e** Bar graph and representative flow plots depicting the significant differences in the frequency of the SARS-Cov-2 spike-specific CD8 + T cells concomitantly producing both IFN-γ and TNF-α. Bars represent the mean + SD and significant differences between the groups were calculated using nonparametric two-tailed Mann–Whitney *U*-test in Graphpad prism version 8. **f** CD8 + T cell polyfunctionality: Differences in the ability of CD8 + T cells stimulated with SARS-CoV-2 spike peptides to secrete more than one cytokine. Boolean gating was applied to identify all combinations of CD8 + T cell effector functions. The pie chart depicts the average proportion of spike-specific CD8 + T cells producing all three (IFN-γ, IL-2, or TNF-α), any two, or any one cytokine. The graphs show cytokine-secreting peptide-specific CD8^+^ T cells for each individual mouse (*n* = 5 /group) in response to each adjuvant formulation and the horizontal line represents the mean. This analysis was performed using the SPICE software version 5.1^65^.

We next enumerated the frequency of antigen-specific CD8^+^ T cells with MHC class I tetramer staining. Epitope mapping data revealed SARS-CoV-2 spike aa 539–546 (VNFNFNGL) as the most immunogenic epitope present in peptide pools 4, 5, and 19 (Fig. 6b, c). MHC class I, K^b^, tetramers presenting the VNFNFNGL epitope (aa 539–546) along with two other H2-K^b^ restricted epitopes, GNYNYLYRL (aa 433–441) and YNYLYRLF (aa 435–443) from the spike protein, were used to identify antigen-specific CD8^+^ T cells. A H2-K^d^ restricted tetramer, containing CYGVSPTKL (aa 365–373) from the spike protein served as a control (Supplementary Table 6). The gating strategy is provided in Supplementary Fig. 6a, b. SpFN + ALFQ generated significantly higher K^b^-spike _(539-546)_-specific CD8^+^ T cells (*p* = 0.01) ranging from 2.47 to 28% (Fig. 7a) compared to 0.21 to 4.2% for SpFN + AH (Fig. 7b). Based on the tetramer and epitope mapping data, we performed combined ICS and tetramer staining experiments (the gating strategy and the Boolean gating is shown in Supplementary Fig. 4) with the K^b^-spike _(539-546)_-specific tetramer, to demonstrate cytokine secretion by tetramer-positive CD8^+^ T cells. SpFN + ALFQ vaccinated mice generated higher percentages of K^b^-spike _(539–546)_-specific TNF-α^+^ (Fig. 7c; *p* = 0.007), IFN-γ^+^ CD8^+^ T cells (Fig. 7d; *p* = 0.008), and double-positive TNF-α^+^IFN-γ^+^ CD8^+^ T cells (Fig. 7e; *p* = 0.007), compared to SpFN + AH vaccinated mice following the booster vaccination.

Cytokine polyfunctionality is of major importance for the successful efficacy of a vaccine^47^. Several studies have shown a strong correlation between the protection level and the induction of high frequencies of polyfunctional T cells co-expressing IFN-γ, TNF-α, and IL-2^47^. To determine if SpFN + ALFQ generated antigen-specific polyfunctional CD8^+^ T cells, we assessed their frequency post booster vaccination. Functionality was measured based on stimulation with the S1 peptide pool, as our prior data showed minimal responses to the S2 peptide pool by ICS.

SpFN + ALFQ induced a strikingly higher percentage of polyfunctional spike-specific CD8^+^ T cells co-expressing either three (IFN-γ, IL-2, and TNF-α) or two (IFN-γ and TNF-α) cytokines compared to the SpFN + AH (Fig. 7f). Polyfunctional IFN-γ and TNF-α producing T cells dominated the response from the SpFN + ALFQ vaccinated animals, in contrast to the single cytokine-secreting T cell response exhibited by the SpFN + AH group. These data demonstrate the differences in the landscape of T cell responses induced by the two different vaccine formulations.

### Prime-boost with SpFN + ALFQ resulted in a large population of a durable spike_(539–546)_-specific memory CD8^+^ T cells in spleens and in perfused lungs

**Fig. 8:**
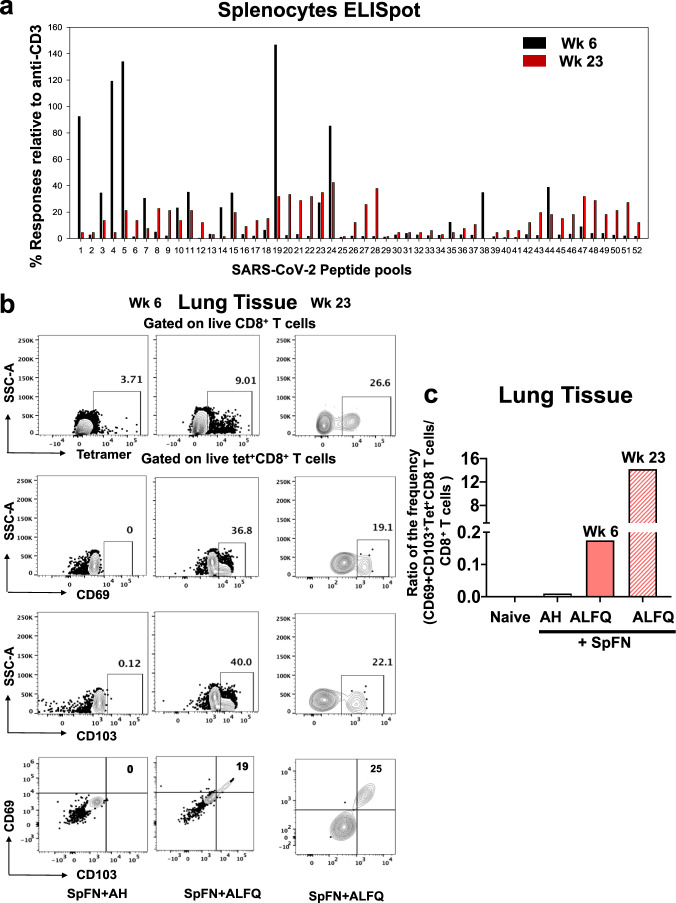
ELISpot Responses in splenocytes and SARS-CoV-2K^b^ spike _(539–546)_-specific MHC class I restricted CD8^+^ T cells in perfused lungs of SpFN prime-boost vaccinated mice at weeks 6 and 23. **a** Number of spike-specific IFN-γ spot forming units in week 6 and week 23 splenocytes stimulated with 52 peptide pools from the Epitope Mapping Peptide Set of the spike protein. The data were presented as % response relative to a number of spots produced with anti-CD3. **b** Frequency of SARS-CoV-2 spike _**(539–546)**_**-**specific tetramer-positive CD8^+^ T cells in lung tissue of SpFN + AH and SpFN + ALFQ vaccinated mice at weeks 6 and 23 as determined by flow cytometry. SARS-CoV-2 spike _**(539–546)**_**-**specific tetramer staining was followed by CD69 and CD103 staining. A representative figure is shown for each week. **c** The ratio of the frequency of CD69^+^ CD103^+^ Tet^+^CD8^+^ T cells to CD8^+^ T cells is shown for Naïve (unimmunized), SpFN + AH at week 6, and SpFN + ALFQ at weeks 6 and 23 post-first immunization.

Based on the results of the ELISpot assay (Fig. 6), we examined if the measured reactivity to the peptide pools were durable. Therefore, splenocytes from SpFN + ALFQ vaccinated mice at week 23 were analyzed with the 52 peptide pools composed of 315 peptides by an IFN-γ-specific ELISpot assay. The reactivity to pools 4, 5, and 19 (Fig. 8a) were greatly diminished but still maintained at week 23. In general peptide pools that showed reactivity at week 6 also showed reactivity at week 23 albeit at a lower magnitude. We noticed a broadening in the responses at week 23 as more peptide pools showed reactivity compared to week 6 especially within the C-terminal region of the spike protein.

Memory T cells responses were further examined in perfused lungs at similar time points as the mediastinal LNs and spleen. At weeks 6 and 23 following primary vaccination (3- and 20-weeks following booster vaccination), perfused lungs (to reduce circulating T cells) were processed from both groups of vaccinated mice and stained with K^b^-spike_(539–546)_ tetramer. Tetramer-positive CD8^+^ T cells were detected from the perfused lungs of mice in both adjuvant groups (Fig. 8b, c). However, only the cells from perfused lung homogenates of SpFN + ALFQ vaccinated mice exhibited CD69 and CD103 expression, two markers of tissue residence. Similar to the results from splenocytes, the lungs from the SpFN + ALFQ group exhibited a higher percentage of K^b^-spike_(539–546)_-specific CD8^+^ T cells (9.01%) compared to lungs from the SpFN + AH group (3.71%). To further analyze the memory response, we also characterized the K^b^-spike_(539–546)_-specific CD8^+^ T cells at 23 weeks post-vaccination (Fig. 8b and Supplementary Fig. 7). We identified K^b^-spike_(539–546)_-specific CD8^+^ T cells in the lung homogenates, which also expressed CD69 and CD103 (Fig. 8b). The gating strategy is shown in Supplementary Fig. 7. The ratio of the frequency of CD69^+^ CD103^+^Tet^+^CD8^+^ T cells to CD8^+^ T cells from the perfused lung tissue of vaccinated mice is shown in Fig. 8c. There was an increase in the frequency of these cells at week 23 compared to week 6 in the lungs of SpFN + ALFQ vaccinated mice. These results would strongly suggest the durability of these memory CD8^+^ T cells both in the spleen and in the lungs.

### K^b^-spike_(539–546)_-specific CD8^+^ T cells from SpFN + ALFQ immunized mice effectively lyse target cells

**Fig. 9:**
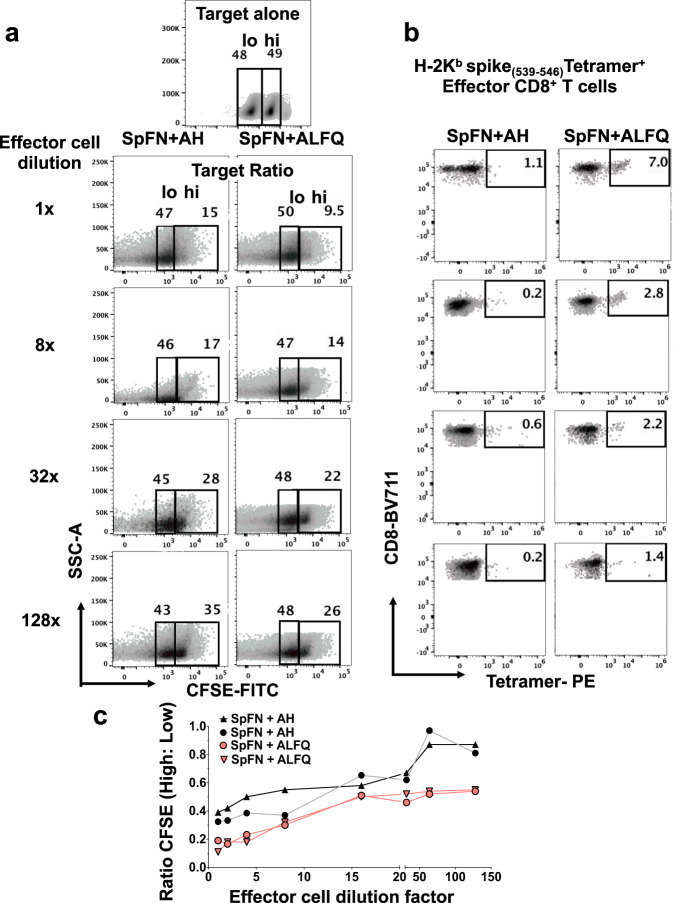
Flow cytometry-based CFSE cytotoxic T cell (CTL) assay to measure antigen-specific killing in response to SpFN vaccine at week 6 following prime-boost vaccination. Flow cytometry plots depicting the frequency of **a** CFSE-high/CFSE-low population and **b** effector cells from the spleens of mice vaccinated with SpFN vaccine adjuvanted with ALFQ or AH. **c** Graph depicting the ratio of CFSE high to low (indicating antigen-specific killing) as a function of effector cell dilution. Each curve represents effector cell dilutions from a single mouse vaccinated with SpFN + ALFQ or SpFN + AH. The curves were evaluated using the Pearson correlation coefficient. Two independent experiments were performed and data from one of the experiments is shown.

To determine if the induction of a more robust CD8^+^ T cell response in the SpFN + ALFQ vaccinated mice translated into improved effector function, we conducted an in vitro killing assay (Fig. 9). Target cells were generated by pulsing naïve splenocytes with K^b^-spike_(539–546)_- peptide or left unpulsed and labeled CFSE high or low, respectively (Fig. 9a, b). The gating strategy for target CFSE-high and low and tetramer-positive effector CD8^+^ T cells is shown in Supplementary Fig. 6c. Effector cells from spleens of SpFN + ALFQ vaccinated mice demonstrated increased killing of peptide-pulsed target cells compared to SpFN + AH group represented by the ratio of CFSE high:low cells at each dilution (Fig. 9c). Increased CTL activity has been linked with reduced viral titers and inhibition of viral replication^48^ and is the central function of the CD8^+^ T cells we identified in this study. Flow plots further demonstrated an increase in the CFSE high population as the dilution of effector cells increased (Fig. 9a). K^b^-spike_(539–546)_- specific CD8^+^ T cells present at each dilution were measured. The decrease in cells from the SpFN + ALFQ vaccinated mice corresponded with a decrease in CFSE high cell death (Fig. 9b). Even at a high dilution rate, the cells from SpFN + ALFQ vaccinated animals demonstrated improved cytolytic activity compared to the AH group. Curves for both ALFQ and AH groups were evaluated using Pearson correlation and showed strong positive correlations of 0.823 and 0.888, respectively (Fig. 9c).

## Discussion

In the present study, we demonstrate early and robust engagement of APCs and vaccine recruited polyfunctional SARS-CoV-2 spike-specific T cells, associated with the development of spike cytolytic memory CD8^+^ T cells induced by the immunogen, SpFN, combined with a potent adjuvant, ALFQ. Based on the breadth of the observed immune response with SpFN + ALFQ, and protection following challenge with USA-WA1/2020, B1.1.7 (Alpha) and B.1.351 (Beta) strains in Syrian golden hamsters and nonhuman primates^31,32^, and the induction of broad SARS coronavirus immune responses in mice^23^, a detailed study of the cellular immune responses induced by SpFN + ALFQ was warranted. The mouse system offers the advantage of defining the in vivo mechanisms of innate and adaptive immunity following vaccination with SpFN + ALFQ in comparison with a common and well-established adjuvant AH.

Following vaccination with SpFN + ALFQ, we observed robust and sustained engagement and activation of classical and nonclassical APC subsets in mechanically dissociated^49,50^ dLNs cells, compared to the AH formulated vaccine. Importantly, we show that cDC1 and cDC2, which are critical for antigen-specific CD8^+^ and CD4^+^ T cell differentiation, exhibited enhanced upregulation of costimulatory molecules necessary for T cell engagement. Vaccines formulated with AS01, which contain similar components to ALFQ, have been shown to depend on DC activation and more recently cDC2s, in particular, to induce antigen-specific T cells^51^. By multiparameter spectral flow cytometry, we were able to analyze the complexity of the early immune response to SpFN + ALFQ in the dLN. Our data indicated that SpFN + ALFQ also recruited more nonclassical APCs and monocytes that support T cell and B cell development capable of enhancing vaccine efficacy^52^. We identified MoDC in the dLN of SpFN + ALFQ vaccinated group, with the potential for cross-presentation of antigen, which may add to the findings of Welsby et al.^53^, who identified MoDC with the potential for antigen presentation in response to AS01. Furthermore, we characterized LN resident macrophage subsets, which have been shown to play a role in vaccine responsiveness^54,55^. MCM increased in number and functional activation in response to SpFN + ALFQ and may support and optimize plasma cell and T cell development as shown by others^56^. In addition, we found that SSM were reduced in frequency upon vaccination with SpFN + ALFQ, which is consistent with the findings of Detienne et al.^37^, regardless of the differences in tissue dissociation methods utilized in the two studies. Although the mechanism of action of saponins is not fully understood, the findings of Detienne et al.^37^ also showed that QS-21 was taken up by SSM upon vaccination and that SSM were essential for the activation of DCs. Prior publication on the combination of MPL and QS-21 in the form of AS01 demonstrate a shorter duration of innate immune activation from 0–2 days^37,51,53^, whereas SpFN + ALFQ exhibited APC engagement and activation through 5 days post-vaccination potentially indicating that the lipid formulation or differences in dosing of this unique adjuvant can extend the duration of antigen presentation. Together our findings suggest that macrophage subsets within the LN such as SSM may support the presentation of vaccine-derived antigen either directly or indirectly through DCs. cDC1 and cDC2 are most effective at presenting antigen to T cell subsets and are frequently targeted in vaccine design. Our data also suggest that additional potential antigen-presenting cells, such as MoDCs, may support the robust T cell and antibody response demonstrated by SpFN + ALFQ. The complex interplay of APCs over 5 days in the dLN that is induced by the unique formulation of SpFN + ALFQ is associated with enhanced antigen-specific T and B cell engagement and memory development and our data support further mechanistic investigations including in vivo studies of vaccine-induced immune cell trafficking to the spleen and other lymphoid organs.

By employing a strategy of sampling immunologically relevant tissues temporally and spatially proximal to vaccination, we show that the SpFN + ALFQ vaccine potentiates innate sensing and mobilizes the cellular drivers of a multifactorial immune response, in particular CD4^+^ T cell responses and durable effector-memory functional CD8^+^ T cell responses at prime tissue site relevant to reduce viral entry. SpFN + ALFQ vaccine elicits a favorable cellular immune response and complements the observed strong breadth and maintenance and inhibition of ACE-2 binding and neutralizing antibody responses in immune sera against variants of concern^23,32^. The frequency of cytokine-positive cells was generally higher in the CD8^+^ T cell population than the CD4^+^ T cell population in the spleens of SpFN + ALFQ vaccinated mice, with robust induction of IFN-γ-producing CD8^+^ T cells. The expression of IFN-γ by these memory T cells is particularly of consequence since it is a key cytokine for several antiviral responses^57,58^. IFN-γ^+^ SARS-CoV-2-specific CD8^+^ T cells have been reported in a majority of patients who recovered from COVID-19 infection^59^. This cytokine has been shown to act in synergy with type I interferons to inhibit the replication of SARS-CoV^60^.

SpFN + ALFQ induced significantly greater numbers of polyfunctional spike-specific CD8^+^ T cells co-expressing either 3 (IFN-γ, IL-2, and TNF-α) or 2 (IFN-γ, TNF-α) cytokines compared to the SpFN + AH. We identified 11 SARS-CoV-2 spike-specific T cell epitopes in spleens of mice vaccinated with SpFN + ALFQ. The most dominant and immunogenic SARS-CoV-2 spike epitope begins in the RBD domain (VNFNFNGL; aa 539–546) and was confirmed as an MHC class I restricted, K^b^(_539–546_) spike-specific CD8^+^ T cell epitope with tetramer staining. These responses were durable and could be measured at week 23, however, the magnitude of the response was higher at week 6 while the breadth increased at week 23. This study reports the expansion of vaccine-induced SARS-CoV-2K^b^-spike(_539–546_) -specific polyfunctional CD8^+^ T cells in mice that also exhibited increased killing of peptide-pulsed target cells in an in vitro cytotoxic T cell assay. Since this epitope is also present in SARS-CoV, the generation of cross-reactive protective T cells against other coronavirus strains and variants warrants further investigation. In addition, the molecular mechanisms involved in early and sustained induction of distinct aspects of the innate immune response resulting from ALFQ vaccine formulation, which then influences adaptive immune responses to remain to be explored.

The highly activated and extended engagement of innate immune cells was accompanied by a more robust and rapid differentiation of effector memory-like cells than seen with AH. This early differentiation of SARS-CoV-2-specific memory-like CD4^+^ and CD8^+^ T cells was associated with increased T_EM_ and T_CM_ cells identified in the mediastinal lymph nodes 6 weeks post-vaccination, and T_RM_-like CD8^+^ T cells identified in the lungs at 23 weeks post-vaccination. Preferential expression of cytokines such as IL-10^61^ or homing receptors such as CXCR6^62^ on T cells induced by adjuvants could facilitate the migration of these cells to specific target tissues. However, the association of adjuvants and trafficking of immune cells needs further investigation. A threefold increase in MHC class I restricted K^b^-spike_(539–546)_-specific CD8^+^ T cells was also observed in the perfused lungs of mice vaccinated with SpFN + ALFQ. Unlike cells from lungs of SpFN + AH vaccinated mice, the MHC class I restricted K^b^-spike_(539–546)_-specific CD8^+^ T cells from SpFN + ALFQ vaccinated mice also expressed both markers of tissue residence, the activation marker CD69 and the α-chain (CD103) of the integrin αEβ7 receptor, not only at 6 weeks post-primary vaccination but also at 23 weeks post-primary vaccination, thereby demonstrating the presence of a strong and durable memory response. The importance of T cell surveillance in the lungs cannot be overstated as this is the primary target site of SARS-CoV-2 infection. The early induction and persistence of CD4^+^ and CD8^+^ T cells may confer long-lasting memory against coronaviruses and induce durable immune responses as was observed in some SARS-CoV survivors, in whom CD4^+^ and CD8^+^ T cells persisted for 17 years^63^.

In conclusion, our study revealed that the adjuvant formulation ALFQ combined with a SARS-CoV-2 spike ferritin nanoparticle vaccine was effective in the early recruitment and activation of multifaceted APCs. The engagement of innate immune cells was associated with potent antigen-specific polyfunctional cytokine responses and cytolytic function. The formulation and composition of ALFQ may regulate the expression of cytokines and homing receptors to facilitate the migration of effector cells to target cells as seen by the presence of long-lived SARS-CoV-2-specific memory CD8^+^ T cell responses in the lung tissue. Together, these findings highlight the importance of the adjuvant ALFQ in orchestrating the interplay of innate and adaptive immune responses.

## Methods

### Mice vaccination and tissue processing

Female C57BL/6 mice (5–6 weeks of age) were obtained from The Jackson Laboratory. Animals were housed in groups and fed standard chow diets. Animal studies were carried out in accordance with the recommendations in the Guide for the Care and Use of Laboratory Animals of the National Institutes of Health. The protocols were approved by the Institutional Animal Care and Use Committee at the Walter Reed Army Institute of Research [Assurance number D16-00596 (A4117-01)]. Mice were vaccinated by intramuscular injection with SpFN + AH (*n* = 5 mice/time point) or SpFN + ALFQ (*n* = 5 mice/time point). Mice received either a single vaccination in the left hind leg quadriceps muscle or the priming vaccination followed by a boost at week 3 in the right hind leg quadriceps muscle. Mice were euthanized on days 3, 5, 7, and 10 post-first vaccination or 3 or 20 weeks after the second vaccination (weeks 6 and 23 post-first vaccination, respectively). Time points 3, 5, and 10 days were repeated twice, each time with an *n* = 5 mice/time point/adjuvant. The 6-week time point with SpFN + ALFQ vaccine was repeated twice (*n* = 5, *n* = 9). Pre-vaccination peripheral blood was obtained on day 4. The dLNs and spleen were collected on days 3, 5, 7, and 10 following the first vaccination. Perfused lung tissues were collected at the 6- and 23-week time points. Mediastinal lymph nodes and spleens were also obtained at the 6-week time point.

The inguinal and popliteal lymph nodes draining the vaccinated leg were combined for analysis. Lymph nodes were manually homogenized between the frosted ends of glass slides to obtain a single-cell suspension. Mononuclear cells were isolated by centrifugation over Fico/Lite-LM (Atlanta Biologicals) density gradient. Single-cell suspensions were washed and resuspended in PBS with 2% FBS. Individual mouse spleens were pressed through a 70-μm cell strainer using the plunger of a 3-mL syringe, to obtain a single-cell suspension, followed by washing twice in PBS containing 2% fetal bovine serum (FBS, Invitrogen). Cells were then counted using trypan blue exclusion, either used immediately or cryopreserved in freezing media (90% heat-inactivated FBS and 10% dimethyl sulfoxide), and stored in liquid nitrogen until use.

### Protein and liposome vaccine formulation

SARS-CoV-2 prefusion antigen construct, derived from the Wuhan-Hu-1 genome sequence was designed as ferritin-fusion recombinant protein for expression as a nanoparticle. The *Helicobacter pylori* ferritin molecule was linked to the C-terminal region of the pre-fusion stabilized ectodomain (residues 12-1158). Several modifications were introduced to stabilize spike trimer formation on the ferritin molecule. The spike ectodomain was modified to introduce two prolines residues (K986P and V987P) as previously described^24^, a “GSAS” substitution at the furin cleavage site (residues 682–685), and increase the coiled-coil interactions of the spike stalk region (residues 1140–1161). The protein was transiently expressed as a soluble recombinant protein in mammalian Expi293 cells (Thermo Fisher Scientific), and the secreted protein self-assembles into a protein nanoparticle, with the SARS-CoV-2 antigen of interest displayed on the nanoparticle surface and was purified by lectin-affinity chromatography and size-exclusion chromatography. A purified protein referred to as spike protein ferritin nanoparticle (SpFN) was formulated in PBS with 5% glycerol at 1 mg/mL and was subsequently diluted with dPBS to provide 10 µg per 50 µL mouse vaccination dose. SpFN immunogen uses 24 copies of antigen spike protein domains supported in a ferritin nanoparticle vaccine platform scaffold that displays the multivalent array on its surface.

Dimyristoyl phosphatidylcholine (DMPC) and dimyristoyl phosphatidylglycerol (DMPG) saturated phospholipids, cholesterol (Chol), and synthetic monophosphoryl lipid A (MPLA, 3D-PHAD^®^) were purchased from Avanti Polar Lipids. DMPC and Chol were dissolved in chloroform, and DMPG and 3D-PHAD^®^ were dissolved in chloroform:methanol (9:1). Alhydrogel^®^, (AH) in a gel suspension was purchased from Brenntag. QS-21 saponin was purchased from Desert King International and was dissolved in Sorensen PBS, pH 6.2 and filtered. For vaccine preparations adjuvanted with ALFQ, lipids were mixed in a molar ratio of 9:1:12.2:0.114 (DMPC:DMPG:Chol: 3D-PHAD^®^), dried by rotary evaporation followed by overnight desiccation, rehydrated by adding Sorensen PBS, pH 6.2, followed by microfluidization to form small unilamellar vesicles (SUV) and filtration^27,64^. QS-21 was added to SUV to form ALFQ (DMPC:DMPG:Chol:MPLA:QS-21; 9:1:12.2:0.114:0.044).

SpFN (600 µg/mL) was mixed with ALFQ (1.5X) in a 1:2 volume ratio. The vial was vortexed at slow speed for 1 min and then put on a roller for 15 min. For vaccine preparations adjuvanted with AH, Alhydrogel^®^ was diluted from a stock concentration of 10 mg/mL to 900 µg/mL (1.5X) with DPBS in a sterile glass vial; SpFN (600 µg/mL) was added in a 1:2 volume ratio to the diluted Alhydrogel^®^. The vial was vortexed at a slow speed for 5 min. The SpFN + ALFQ and SpFN + AH vials were stored at 4 °C for 1 h prior to vaccination and then injected into animals within an hr and stored at 4 °C for 1 h prior to vaccination. Each vaccine dose in 50 μl volume contained 10 μg SpFN, 20 μg 3D-PHAD, and 10 μg QS-21 (ALFQ) or 10 μg SpFN and 30 µg AL^3+^ (AH).

### Flow cytometric phenotyping and intracellular cytokine staining (ICS)

Spectral flow cytometry was performed to analyze APCs and T cells in the lymph nodes as indicated. Single-cell suspensions were treated with Fc Block (BD Biosciences) for 5 min at 4 °C before further staining. Live dead blue dye (Invitrogen) was used to stain dead cells. Analysis of APC and T cell phenotypes were performed using the fluorochrome-conjugated antibodies (Supplementary Table 1). Intracellular cytokine analysis was performed to identify SARS-CoV-2 spike-specific T cells. Single-cell suspensions from the dLNs were plated at 0.5 × 10^6^ cells/well in a 96-well plate and stimulated with the spike (S) 1 or S2 peptide pools from JPT at a final concentration of 1 μg/mL for 6 h at 37 °C in RPMI and 10% FBS. To prevent the secretion of cytokines, GolgiStop (BD Biosciences) was added 1 h after the addition of peptides. Spike-specific T cells were measured by surface staining followed by fixation and permeabilization using BD fixation/permeabilization solution kit (BD Biosciences,) and intracellular cytokine staining (Supplementary Table 1). As negative and positive controls, cells were cultured in media without peptide stimulation or with phorbol 12-myristate 13-acetate (PMA) and ionomycin (BioLegend), respectively. T cells were considered to be responsive to peptide stimulation if the frequency of cytokine-expressing T cells was greater than 0.01% of the parent population after background subtraction. Samples were analyzed on a 5-laser Cytek Aurora flow cytometer (Cytek Biosciences). The data were analyzed using FlowJo software v10 (TreeStar, Inc.). Gating strategy for APC subsets, T cell phenotyping, and ICS are shown in Supplementary Fig. 1a and Fig. 2c, respectively.

Cryopreserved splenocytes were quickly thawed and added to 10 mL of complete RPMI 1640 media supplemented with 5% FBS and 1% Pen-strep followed by viability assessment by trypan blue exclusion method. Approximately, 1 × 10^6^ splenocytes were cultured in the presence of peptide pools directed towards SARS-CoV-2 spike protein S1 or S2 (1 µg/mL) at 37 °C, 5% CO_2_. After 1 h of incubation with peptides, protein transport inhibitor (BD Golgi Plug™ containing Brefeldin A, 1 µg/mL, and BD Golgi Stop™ containing monensin, 1 µg/mL, BD Biosciences) was added for an additional 5 h at 37 °C, 5% CO_2_. For the positive control, cells were stimulated with PMA and ionomycin (Sigma-Aldrich; 50 ng/mL and 1 μg/mL final concentration, respectively) or concanavalin A (positive control for IL-4), while media served as a negative control. After the incubation period, cells were stained with LIVE/DEAD Fixable Aqua Dead Cell Stain Kit (Invitrogen), followed by surface staining with antibodies specific for the following cell surface markers (BUV737-anti-CD3, BUV395-anti-CD4, BV711-anti-CD8 (Supplementary Table 4), obtained from either BD Biosciences, Thermo Fisher Scientific or Biolegend, washed twice with FACS buffer and then fixed/permeabilized for 40 min at 4 °C in the dark using the eBioscience™ Intracellular Fixation and Permeabilization Buffer Set (Thermo Fisher Scientific) as per the manufacturer’s instructions. Cells were then incubated with a panel of intracellular antibodies specific for the following cytokines (V450 anti-IFN-γ, FITC anti-TNF-α, PerCP-Cy5 anti-IL-4, PE anti-IL-2; Table S4) for 30 min at 4 °C, washed twice, and resuspended in FACS buffer. Appropriate single-color compensation controls and Fluorescence minus one (FMO) control were prepared simultaneously and were included in each analysis. Flow cytometric analysis was performed on a BD LSR II flow cytometer, and data were acquired using Diva software (BD Biosciences). The results were analyzed using FlowJo software (TreeStar). In each analysis, respective FMO controls were used to set up the gates or to identify the positive populations. Evaluation of co-expression of different cytokines was performed using the FlowJo Boolean gate platform. The gating strategy applied for the evaluation of flow cytometry-acquired data is provided in Supplementary Fig. 5.

### Measurement of cytokine levels in the serum and spleen cell culture supernatants

Cytokine levels were measured using V-Plex Plus Multi-Spot Assay plates, from Meso Scale Discovery (MSD, Rockville, MD). The mouse pro-inflammatory panel containing IFN-γ, IL-1β, IL-2, IL-4, IL-5, IL-6, KC/GRO (CXCL1), IL-10, IL-12p70, and TNF-α kit included diluent, wash buffer, detection antibody solution, and read buffer, as well as calibrators and controls for each analyte, from the manufacturer. Plates were washed three times with MSD wash buffer before the addition of MSD reference standard and calibrator controls used for quantifying antibody concentrations. Serum samples were diluted 1:2 in MSD diluent buffer, then added to the wells in duplicate. Plates were incubated overnight at 4 °C, then washed three times. MSD detection antibody solution was added to each well, plates were incubated for 2 h at RT with shaking at 500 rpm and washed three times followed by the addition of read buffer T. Plates were read by MESO SECTOR S 120 Reader. Analyte concentration was calculated using DISCOVERY WORKBENCH^®^ MSD Software and reported as pg/mL. For cell supernatants, ~1 × 10^6^ cells were cultured in the presence of peptide pools directed towards SARS-CoV-2 spike protein (1 µg/mL) for 6 h at 37 °C, 5% CO_2_. Cells were centrifuged at 500 × *g* for 10 min and the supernatant was collected. Supernatants were diluted 1:2 in MSD diluent buffer and subsequently processed as described above.

### ELISpot

Multiscreen plates (Millipore) were coated with anti-IFN-γ antibody (capture mAb; 1 µg/mL) according to the manufacturer’s instructions (R&D Systems). Plates were blocked using culture medium (DMEM containing 10% FBS, Pen/Strep, HEPES, NEAA, sodium pyruvate, 2-mercaptoethanol). Thawed splenocytes were plated at 4 × 10^6^ cells/mL (50 μL/well) in triplicate. Recombinant peptides, Epitope Mapping Peptide Set (EMPS), SARS-CoV-2, Spike protein (JPT, 1.25 µg/mL), and anti-CD3 (as positive control) were used to stimulate the cultures. Plates were incubated for 42 h in the presence of antigen at 37 °C and then cells were removed by washing the plates in an automated plate washer. Plates were incubated with biotinylated anti-IFN-γ antibody (1 µg/mL) overnight at 4 °C. Plates were washed and incubated with Streptavidin-alkaline phosphatase for 2 h at RT. The color was developed using ELISpot Blue Color Module (R&D Systems) and processed according to the manufacturer’s instructions. Plates were counted and data analyzed using the AID Autoimmun Diagnostica GmbH ELISpot reader and software. T cell responses were considered positive when the mean spot count exceeded the mean ± 3 SD of the negative control wells.

### Enumeration of SARS-CoV-2 Spike-specific CD8^+^ T cells by tetramer staining

MHC Class I or H-2K^b^ restricted SARS-CoV-2 spike peptide (aa 539–546; VNFNFNGL)-PE-conjugated tetramer (NIH tetramer core facility) was used for the detection of antigen-specific CD8^+^ T cells. (Supplementary Table 2 shows a list of tetramers used in the assay). Approximately, 1 × 10^6^ freshly isolated splenocytes from mice were first stained with a dead cell discrimination dye (Aqua stain, 1:1000 dilution, Invitrogen) for 30 min at 4 °C followed by washing twice with 1XPBS. Cells were blocked with anti-mouse CD16/CD32 for 30 min followed by further incubation with 1 µL (1.2 µg) of PE-conjugated H-2K^b^ SARS-CoV-2 spike-specific tetramer for 30 min at 4 °C in the dark. After washing, cells were further surface stained with BUV737-anti-mouse CD3, BUV395-anti-mouse CD4, BV711-anti-mouse CD8a, and incubated for an additional 30 min in the dark at room temperature, washed twice, and resuspended in FACS buffer. Flow cytometric acquisition and data analysis were performed as described above with appropriate controls. Staining with H-2K^d^ restricted SARS-CoV-2 spike peptide (aa 365–373; CYGVSPTKL)-PE-conjugated tetramer served as a control tetramer in this analysis.

### Detection of SARS-CoV-2 Spike-specific tetramer^+^CD8^+^ T cells in perfused lung tissue

Lungs were perfused with ~7–10 mL of 0.5 mM EDTA in Hanks Balanced Salt Solution. Lungs were cut into small pieces (3–5 mm) and placed in a 15 mL conical tube containing 5 mL RPMI supplemented with 150 U/mL collagenase, 25 U/mL DNase IV (Fisher Scientific), and incubated for 60 min at 37 °C on a rotator at 12–15 RPM. The tissue was then pressed through a 100-μm cell strainer using the plunger of a 5-mL syringe, to obtain a single-cell suspension. Cells were washed twice in RPMI 1640 containing 10% FBS and ~250,000 freshly isolated lung cells from mice were first stained with a dead cell discrimination dye (Live dead Near IR dead cell stain kit, 1:1000 dilution, Invitrogen) for 30 min at 4 °C followed by washing twice with 1XPBS. Cells were blocked with anti-mouse CD16/CD32 for 30 min followed by further incubation with 1 µL (1.2 µg) MHC class I or H-2K^b^ restricted SARS-CoV-2 spike peptide (aa 539–546; VNFNFNGL)-PE-conjugated tetramer for 30 min at 4 °C in the dark. Cells were washed, stained with BUV737-anti-mouse CD3, BUV395-anti-mouse CD4, BV711-anti-mouse CD8a, BV650 anti-CD69, and BV510 anti-CD103, incubated for an additional 30 min in the dark at room temperature, washed twice, and resuspended in FACS buffer. Flow cytometric analysis was performed on a BD LSR II flow cytometer and data were acquired using Diva software (BD Biosciences). The results were analyzed using FlowJo software (TreeStar). Appropriate single-color compensation controls and fluorescence minus one (FMO) control were prepared simultaneously and included. The gating strategy is shown in Supplementary Fig. 7.

### Detection of SARS-CoV-2 spike-specific (Tetramer-positive) CD8^+^ T cells secreting cytokines by flow cytometry

To detect the epitope-specific CD8^+^ T cell cytokine production, we combined antigen stimulation with tetramer labeling and intracellular cytokine staining. Splenocytes (2 × 10^6^/well) were cultured in a 96-well v-bottom plate in the presence of peptide pools directed towards SARS-CoV-2 spike protein S1 (1 µg/mL) at 37 °C, 5% CO_2_. After 1 h of incubation with peptides, protein transport inhibitor (BD Golgi Plug™ containing Brefeldin A, 1 µg/mL, and BD Golgi Stop™ containing monensin, 1 µg/mL) was added followed by an additional 5-h incubation at 37 °C, 5% CO_2_. Cells were washed at 300 g at 4 °C for 7 min, labeled with a dead cell discrimination dye for 30 min at 4 °C followed by washing twice with 1XPBS. Cells were fixed/permeabilized for 40 min at 4 °C in the dark using the eBioscience™ Intracellular Fixation and Permeabilization Buffer Set as per the manufacturer’s instructions. Cells were then blocked for 20 min at 4 °C in Fc-blocking solution (5 μg/mL of CD16/CD32 mAb) and further stained for 40 min at RT with 1 µL (1.2 µg) MHC class I or H-2K^b^ restricted SARS-CoV-2 spike peptide (aa 539–546; VNFNFNGL)-PE-conjugated tetramer (NIH tetramer core facility) diluted in Perm/Wash buffer. In the last 20 min of tetramer incubation, the following mix of fluorescent antibodies were added: BUV737-anti-mouse CD3, BUV395-anti-mouse CD4, BV711-anti-mouse CD8a, and antibodies directed towards intracellular cytokines such as V450 anti-IFN-γ, FITC anti-TNF-α, and APC anti-IL-2 (Supplementary Table 4), then washed twice and resuspended in FACS buffer. Appropriate single-color compensation controls and Fluorescence minus one (FMO) control were prepared simultaneously and were included in each analysis. Flow cytometric acquisition and data analysis were performed as described above with appropriate controls.

### Cytotoxic T lymphocyte killing assay

Single-cell suspensions from spleens of vaccinated mice were used as effector cells and naive spleens were used as target cells. Target cells (20 × 10^6^/mL) were suspended in 2 × 500 µL aliquots (10 × 10^6^ each). One aliquot was incubated in the presence of SARS-CoV-2 spike protein-specific peptides LVKNKCVNFNFNGLT and KCVNFNFNGLTGTGV at 1 µg/mL (JPT) and the other aliquot was incubated in media for 45 min in a 37 °C water bath. Both aliquots were washed twice with 3 mL PBS. CFSE high and low solutions were made at 0.5 and 0.05 µM, respectively using Thermo Fisher CellTrace™ CFSE Cell Proliferation Kit. Peptide pulsed and non-pulsed cells were resuspended respectively in CFSE high and low solutions, incubated for 10 min in a 37 °C water bath, and then washed twice in media. Target cells were resuspended at 2 × 10^6^/mL and 50 µL of each CFSE high and low (1:1, CFSE high:CFSE low) was added to each well. Effector cells were resuspended at 40 × 10^6^/mL (10 × 10^6^ cells in 250 µL) and twofold serial dilutions were made by adding 125 µL of cells to 125 µL of media. About 100 µL of each dilution was added to a well containing the target cell mixture, bringing the total volume to 200 µL. Cells were co-incubated overnight at 37 °C, 5% CO_2_. Following incubation, cells were stained with Live/Dead Aqua stain (1:1000 dilution) for 30 min at 4 °C followed by washing twice with 1XPBS. Cells were blocked with anti-mouse CD16/CD32 for 30 min followed by further incubation with 1 µL (1.2 µg) of PE-conjugated H-2K^b^ spike_(539–546)_-specific tetramer for 30 min at 4 °C in the dark. After washing, cells were further stained with BUV737-anti-mouse CD3, BUV395-anti-mouse CD4, BV711-anti-mouse CD8a, and incubated for an additional 30 min in the dark at room temperature. Flow cytometric acquisition and data analysis were performed as described above with appropriate controls.

### Statistical analysis

Flow cytometric data were analyzed using FlowJo v.10.0.8 (BD). Data were displayed as dot plots or bar graphs. AH and ALFQ groups were compared by unpaired Student’s *t*-test or Mann–Whitney *U*-test or Pearson correlation. Graphs were plotted using GraphPad Prism v.8.4.0. Statistical analyses were conducted using GraphPad Prism v.8.4.0 software. *P* values <0.05 were considered statistically significant.

### Reporting Summary

Further information on research design is available in the Nature Research Reporting Summary linked to this article.

## Supplementary information

Supplementary Information

Reporting Summary

## Data Availability

All the data supporting the findings of this study can be found within the paper in Figs. 1–9 and in Supplementary Data Figs. 1 to 7. The spike (S) protein sequence was derived from the Wuhan-Hu-1 genome sequence (GenBank accession number: MN908947.3).
