# Supplementary material for: SARS-CoV-2 ferritin nanoparticle vaccine induces robust innate immune activity driving polyfunctional spike-specific T cell responses
Source: NPJ Vaccines. 2021 Dec 13;6:151. doi: 10.1038/s41541-021-00414-4 (PMC8668928; doi:10.1038/s41541-021-00414-4)
Supplement: Supplementary file 1 — Supplementary Information [file 41541_2021_414_MOESM1_ESM.pdf]

## Supplementary Materials

# SARS-CoV-2 ferritin nanoparticle vaccine induces robust innate immune activity driving polyfunctional spike-specific T cell responses

Joshua M. Carmen[1]*, Shikha Shrivastava[1,2]*, Zhongyan Lu[3]*, Alexander Anderson[1,2], Elaine B. Morrison[1], Rajeshwer S. Sankhala[4,5], Wei-Hung Chen[4,5], William C. Chang[4,5], Jessica S. Bolton[6], Gary R. Matyas[1], Nelson L. Michael[7], M. Gordon Joyce [4,5], Kayvon Modjarrad[4], Jeffrey R. Currier[8], Elke Bergmann-Leitner[6], Allison M.W. Malloy[3†], and Mangala Rao[1‡]

[‡]Corresponding author. Email: mrao@hivresearch.org
[†]Co-Corresponding Author: Email: Allison.malloy@usuhs.edu

This file includes:

## SUPPLEMENTARY MATERIALS

**Supplementary Fig. 1.** Flow cytometry analysis of innate immune cells in draining lymph nodes responding to SpFN vaccine adjuvanted with ALFQ or AH.

**Supplementary Fig. 2.** Characterization of the responding T cells upon priming with SpFN vaccine adjuvanted with ALFQ or AH.

**Supplementary Fig. 3.** Frequency of total CD3$^+$ T cells in splenocytes of mice following prime and prime boost vaccination with SpFN adjuvanted with AH or ALFQ.

**Supplementary Fig. 4.** Flow gating strategy to determine the frequency of cytokine secreting CD4$^+$ and CD8$^+$ T cells.

**Supplementary Fig. 5.** Amino acid sequence of the SARS-COV-2 spike protein.

**Supplementary Fig. 6**. Flow gating strategy to determine the frequency of SARS-CoV-2 spike-specific CD8$^+$ T cells in the splenocytes of mice as detected by MHC class I tetramer staining following SpFN vaccination.

**Supplementary Fig. 7.** Flow gating strategy to determine the frequency of SARS-CoV-2 spike-specific CD8$^+$ T cells in perfused lungs of SpFN vaccinated mice as detected by MHC class I tetramer staining followed by CD69 and CD103 staining.

**Supplementary Table 1**. List of antibodies used for Cytex flow cytometry-based analysis of the APC phenotypes

**Supplementary Table 2.** List of antibodies used for Cytex flow cytometry-based analysis of the T cell phenotypes

**Supplementary Table 3**. List of antibodies used for Cytex flow cytometry-based analysis of the T cell cytokine staining

**Supplementary Table 4**. List of antibodies used for intracellular cytokine staining by BD LSRII flow cytometry-based analysis of the cytokine response in the splenocytes

**Supplementary Table 5.** List of antibodies used for staining perfused lung cells and analysis by BD LSRII flow cytometry

**Supplementary Table 6**. List of MHC class I tetramer used for the detection of SARS-CoV-2 spike specific $CD8^+$ T cell response

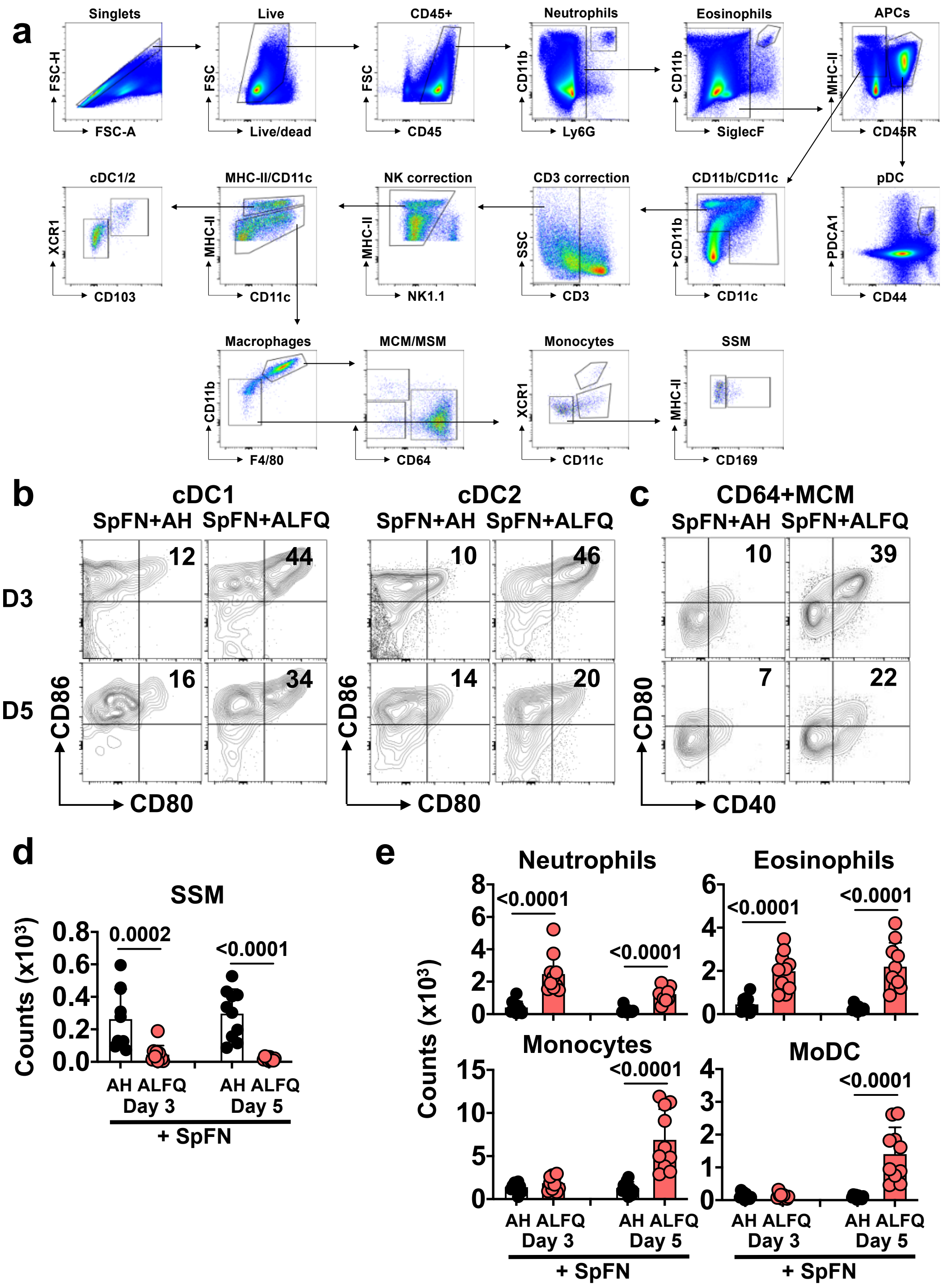


**Supplementary Fig. 1. Spectral flow cytometry analysis of innate immune cells in draining lymph nodes responding to SpFN vaccine adjuvanted with ALFQ or AH.**

**(a)** Flow cytometry gating strategy for the identification of neutrophils (Ly6G$^+$CD11b$^+$), eosinophils (Siglec F$^+$CD11b$^+$), pDCs (MHC-II$^+$CD45R$^+$PDCA1$^+$), cDC1 (MHC-II high CD11c$^+$XCR1$^+$CD103$^+$), cDC2 (MHC-II high CD11c$^+$XCR1$^-$), MSM (MHC II$^+$F4/80$^+$CD11b$^+$CD169$^+$CD64$^-$), MCM (MHC-II$^+$F4/80$^+$CD11b$^+$CD169-CD64+/-), SSM (CD11b$^+$CD169$^+$F4/80$^-$), XCR1+cDC1-like MoDCs (MHC-II+CD11b int CD11c$^+$XCR1$^+$), XCR1-CD11c$^+$MoDCs (MHC-II$^+$CD11b int CD11c$^+$XCR1$^-$), and monocytes (MHC-II$^+$CD11b int CD11c$^-$XCR1$^-$). **(b)** Flow cytometry plots demonstrating expression of costimulatory molecules CD80 and CD86 by cDC upon activation after vaccination. (**c**) Flow cytometry plots demonstrating activation of MCM by expression of CD40 and CD80. **(d)** Magnitude of SSM, and **(e)** other non-classical APCs, including neutrophils, eosinophils, XCR1$^+$MoDCs, and monocytes at days 3 and 5 post priming vaccination. Bars indicate mean $\pm$ s.d.

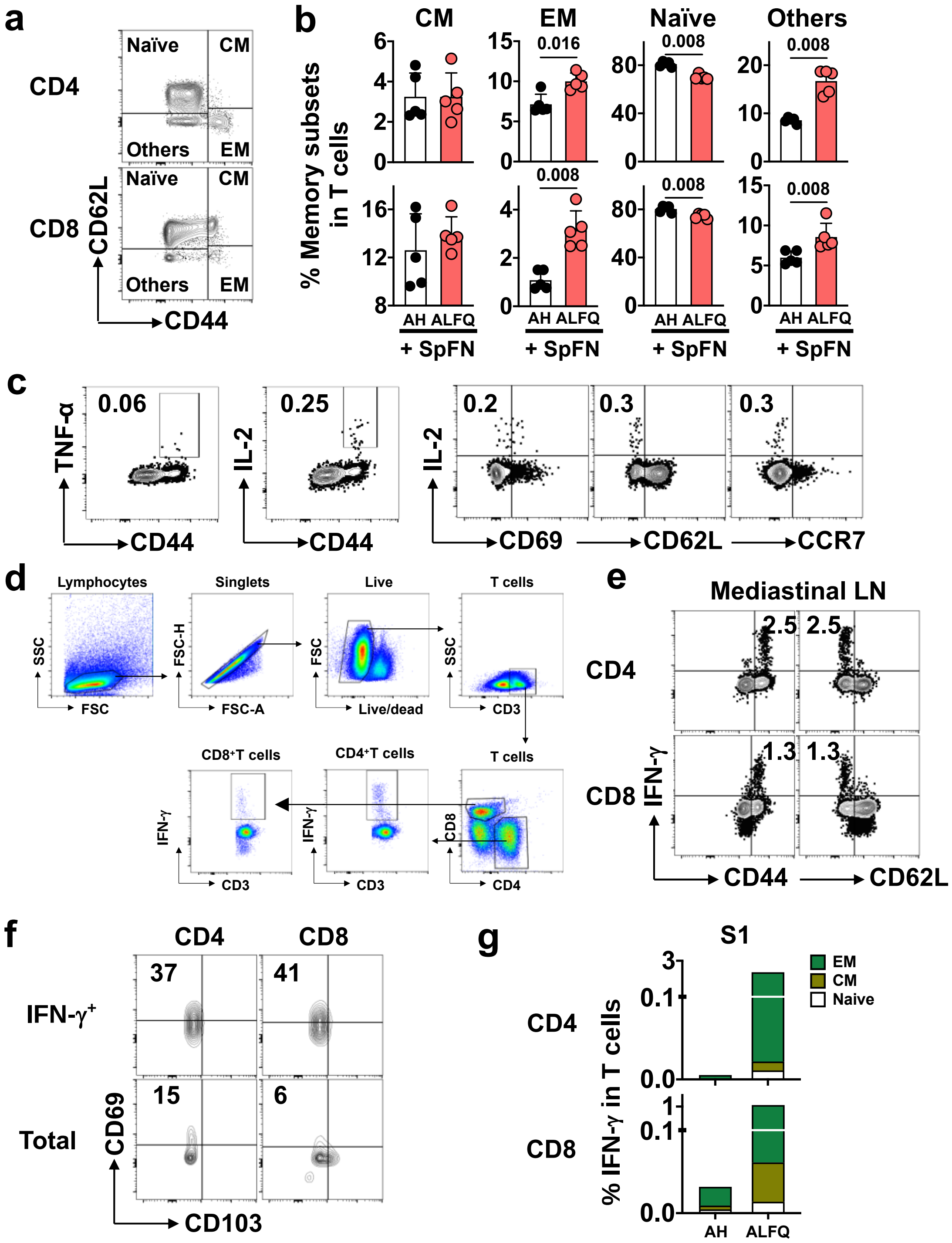


**Supplementary Fig. 2. Characterization of responding T cells in lymph nodes after SpFN vaccination. (a)** Early indication of memory potential phenotypes shown as flow plots (left panels) and quantitation of phenotype potential between the two vaccine groups at day 7 after the first vaccination (right panel) in the dLNs. **(b)** Memory potential was characterized as naive (CD62L$^{+}$CD44$^{-}$), central memory (CD62L$^{+}$CD44$^{+}$), effector memory (CD62L$^{-}$CD44$^{+}$), and other. **(c)** Cytokine expression and activation markers characterizing SARS-CoV-2 spike-specific CD4$^{+}$ T cells at day 10 day after the first vaccination. **(d)** Flow cytometry gating strategy for the identification of SARS-CoV-2 spike-specific CD4$^{+}$ and CD8$^{+}$ T cells in the mediastinal LNs at week 6 post prime-boost vaccination. **(e-f)**, Characterization of the SARS-CoV-2 spike-specific CD4$^{+}$ and CD8$^{+}$ T cells in the mediastinal LNs at week 6 following prime-boost vaccination by the expression of IFNγ. IFNγ$^{+}$T cells are **(e),** CD44$^{+}$CD62L$^{-}$ and **(f)** more frequently CD69$^{+}$ than parent populations, but CD103$^{-}$, indicating activated status. **(g)** Memory phenotype of IFNγ$^{+}$T cells in the mediastinal LN 6 weeks post-vaccination. Bars indicate mean ± s.d. Differences between the two groups were analyzed by using non-parametric Mann–Whitney U-test with $P \leq 0.05$ considered as statistically significant.

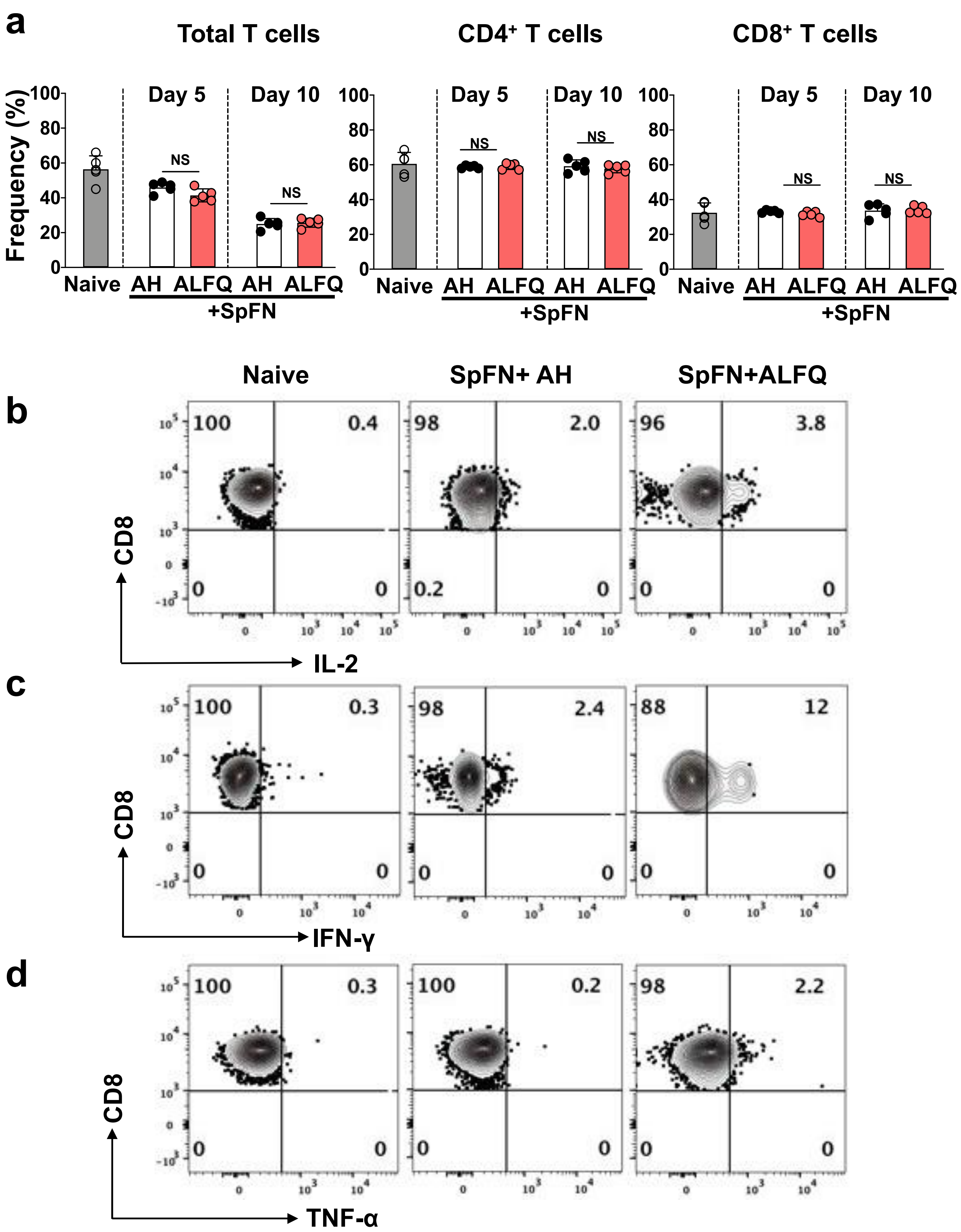


**Supplementary Fig. 3. Frequency of T cells in splenocytes of mice following priming vaccination with SpFN vaccine.** **(a)** Frequency of total $CD3^+$, $CD4^+$ and $CD8^+$ T cells in splenocytes of mice on days 5 and 10 post priming vaccination with SpFN adjuvanted with AH or ALFQ. **(b-d)** Representative flow plots depicting the frequency of **(b)** IL-2 **(c)** IFN-γ and **(d)** TNF-α secreting $CD8^+$ T cells on day 10 post priming vaccination. Bars indicate mean $\pm$ s.d.

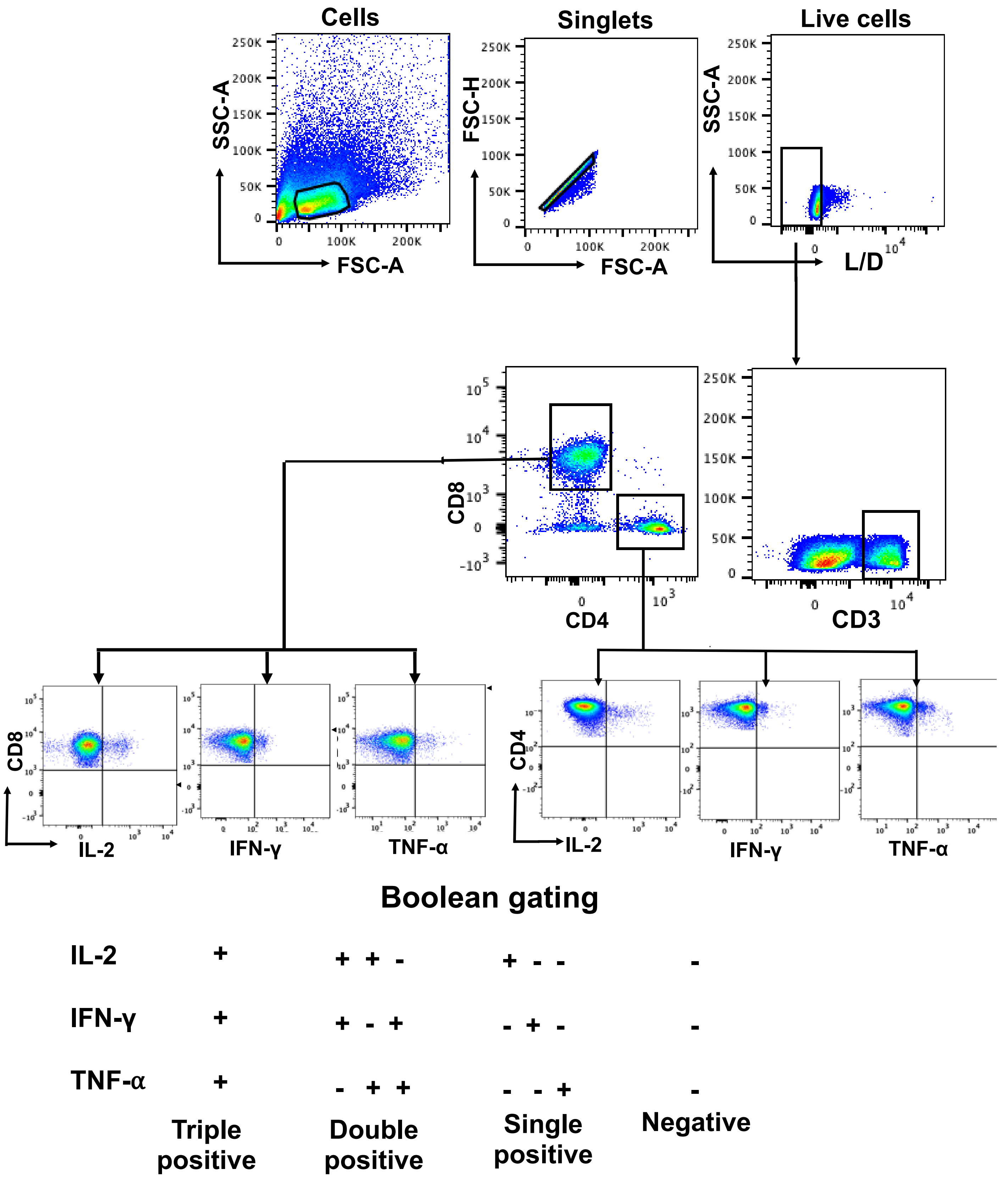


**Supplementary Fig. 4. Flow gating strategy to determine the frequency of cytokine secreting CD4$^+$ and CD8$^+$ T cells.** Flow gating strategy to determine the frequency of cytokine secreting IL-2, IFN-γ, and TNF-α secreting CD4$^+$ and CD8$^+$ T cells. Gates were defined on the respective fluorescence minus one (FMO) control. Boolean gating was performed to identify single, double, and triple positive intracellular cytokine positive cells.

1 59
MFVFLVLLPLVSSQCVNLTTRTQLPPAYTNSFTRGVYYPDKVFRSSVLHSTQDLFLPFF
60 119
SNVTWFHAIHVSGTNGTKRFDNPVLPFNDGVYFASTEKSNIIRGWIFGTTLDSKTQSLLI
120 179
VNNATNVVIKVCEFQFCNDPFLGVYYHKNNKSWMESEFRVYSSANNCTFEYVSQPFLMDL
180 239
EGKQGNFKNLREFVFKNIDGYFKIYSKHTPINLVRDLPQGFSALEPLVDLPIGINITRFQ
240 299
TLLALHRSYLTPGDSSSGWTAGAAAYYVGYLQPRTFLLKYNENGTITDAVDCALDPLSET
300 359
KCTLKSFTVEKGIYQTSNFRVQPTESIVRFPNITNLCPFGEVFNATRFASVYAWNRKRIS
360 419
NCVADYSVLYNSASFSTFKCYGVSPTKLNDLCFTNVYADSFVIRGDEVRQIAPGQTGKIA
420 479
DYNYKLPDDFTGCVIAWNSNNLDSKVGGNYNYLYRLFRKSNLKPFERDISTEIYQAGSTP
480 539
CNGVEGFNCYFPLQSYGFQPTNGVGYQPYRVVVLSFELLHAPATVCGPKKSTNLVKNKCV
540 599
NFNFNGLTGTGVLTESNKKFLPFQQFGRDIADTTDAVRDPQTLEILDITPCSFGGVSVIT
600 659
PGTNTSNQVAVLYQDVNCTEVPVAIHADQLTPTWRVYSTGSNVFQTRAGCLIGAEHVNNS
660 719
YECDIPGAGICASYQTQTNSPRRARSVASQSIIAYTMSLGAENSVAYSNNSIAIPTNFTIS
720 779
VTTEILPVSMTKTSVDCTMYICGDSTECSNLLLQYGSFCTQLNRALTGIAVEQDKNTQEVF
780 839
AQVKQIYKTPPIKDFGGFNFSQILPDPSKPSKRSFIEDLLFNKVTLADAGFIKQYGDCLGD
840 899
IAARDLICAQKFNGLTVLPPLLTDEMIAQYTSALLAGTITSGWTFGAGAALQIPFAMQMA
900 959
YRFNGIGVTQNVLYENQKLIANQFNSAIGKIQDSLSSTASALGKLQDVVNQNAQALNTLVK
960 1019
QLSSNFGAISSVLNDILSRLDKVEAEVQIDRLITGRLQSLQTYVTQQLIRAAEIRASANLA
1020 1079
ATKMSECVLGQSKRVDFCGKGYHLMSFPQSAPHGVVFLHVTYVPAQEKNFTTAPAICHDGK
1080 1139
AHFPREGVFVSNGTHWFVTQRNFYEPQIITTDNTFVSGNCDVVIGIVNNTVYDPLQPELDS
1140 1199
FKEELDKYFKNHTSPDVDLGDISGINASVVNIQKEIDRLNEVAKNLNESLIDLQELGKYEQ
1200 1259
YIKWPWYIWLGFIAGLIAIVMVTIMLCCMTSCCSCLKGCCSCGSCCKFDEDDSEPVLKGVKL
1260
HYT

**Supplementary Fig. 5. Amino acid sequence of the SARS-CoV-2 spike protein.**
Highlighted peptides were part of ELISpot-reactive SARS-CoV-2 glycoprotein derived matrix peptide pools. Underlined peptides are sequences predicted by NETMHC prediction algorithm (iedb.org). Numbers indicate amino acid residues.

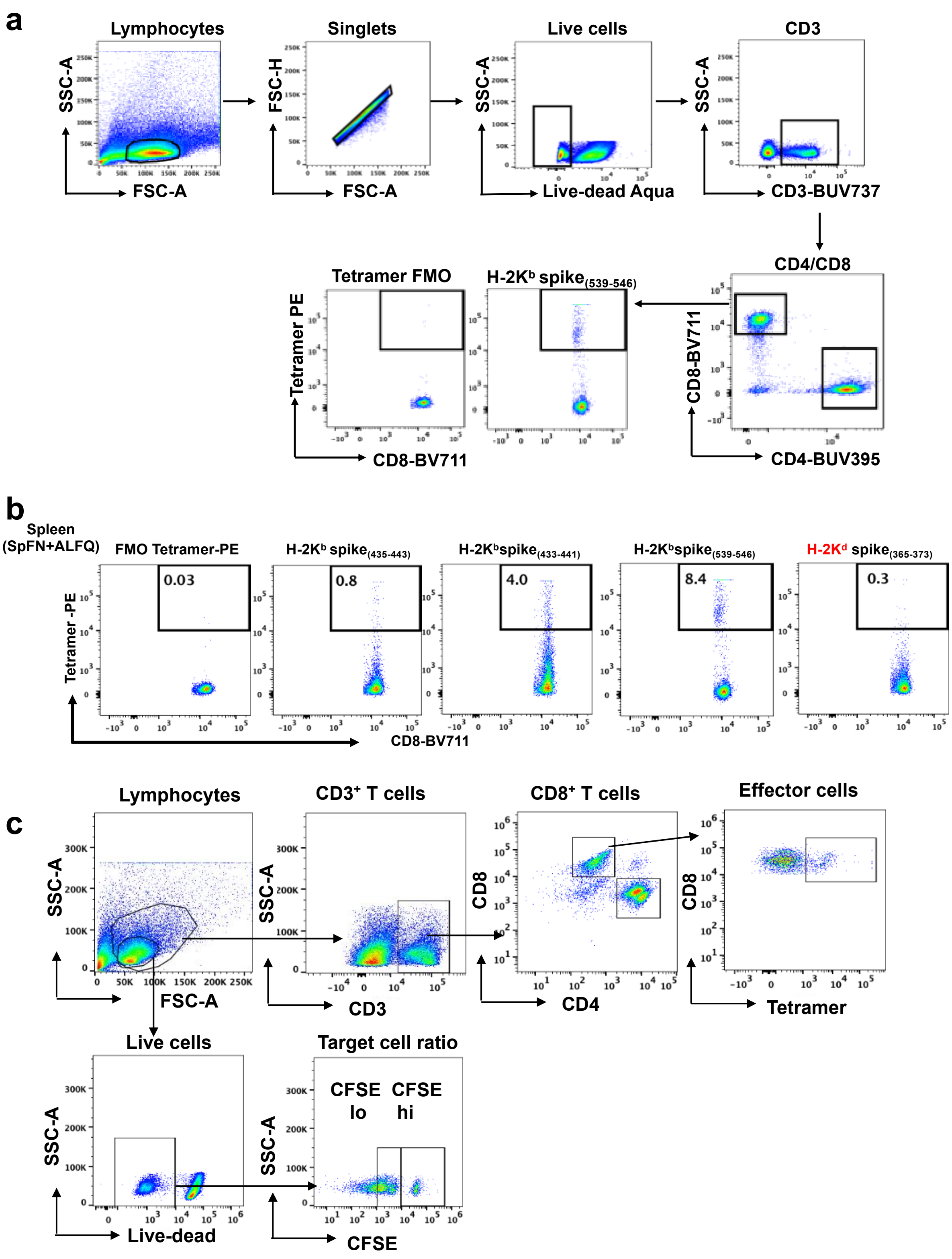


**Supplementary Fig. 6. Flow gating strategy for determining the frequency of SARS-CoV-2 specific CD8⁺ T cells and for the CTL assay following SpFN vaccination**
**(a)** Flow gating strategy to determine the frequency of SARS-CoV-2 spike specific CD8⁺ T cells in the splenocytes of SpFN vaccinated mice as detected by MHC class I tetramer staining. **(b)** Cells were stained for four different tetramers directed towards four different epitopes on SARS-CoV-2 spike protein [$K^b$ $_{(433\text{-}441)}$; $_{(435\text{-}443)}$; $_{(539\text{-}546)}$ and $K^d$ $_{(365\text{-}373)}$]. Detailed information is provided in Supplementary Table 6. **(c)** Gating strategy used to determine the frequency of tetramer positive CD8⁺ T cells (effector cells) and ratio of CFSE high (hi): low (lo) target cells in the cytotoxic T lymphocyte killing assay.

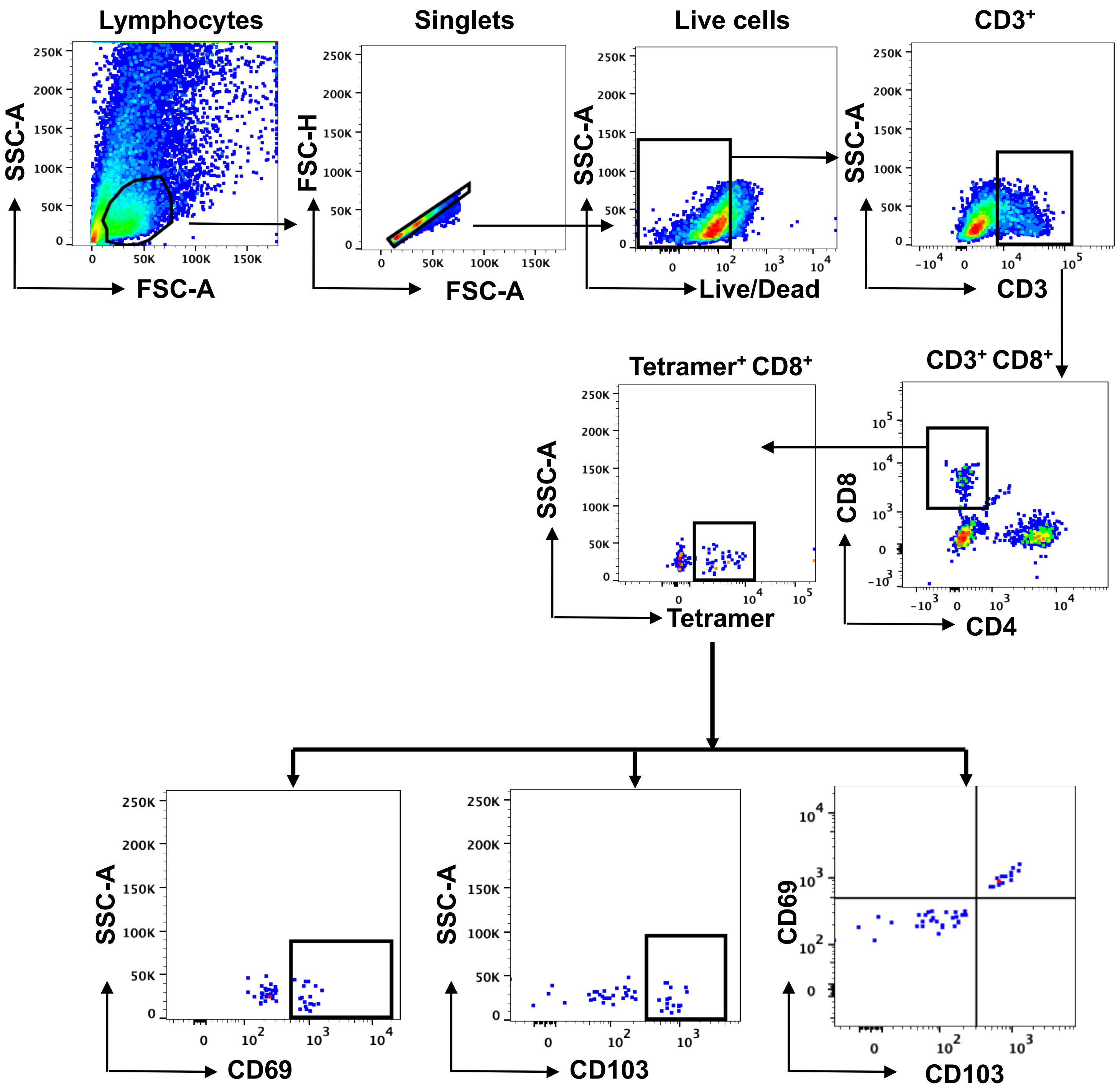


**Supplementary Fig. 7. Flow gating strategy to determine the frequency of SARS-CoV-2 spike-specific CD8$^{+}$ T cells in perfused lungs of SpFN vaccinated mice as detected by MHC class I tetramer staining followed by CD69 and CD103 staining.**
Flow gating strategy to determine the frequency of SARS-CoV-2 spike $_{(539\text{-}546)}$-specific tetramer positive CD8$^{+}$ T cells followed by CD69 and CD103 staining in lung tissue of SpFN vaccinated mice.

**Supplementary Table 1**. List of antibodies used for Cytex flow cytometry-based analysis of the APC phenotypes

| Antigen | Fluorochrome | Clone | Manufacturer | Catalog number |
|---|---|---|---|---|
| CD45R/B220 | BUV395 | RA3-6B2 | BD | 563793 |
| CD80 | BUV661 | 16-10A1 | BD | 741515 |
| CD3 | BUV737 | 145-2C11 | BD | 612771 |
| CD86 | BUV805 | GL1 | BD | 741946 |
| CCR7 | BV421 | 4B12 | BioLegend | 120120 |
| CD40 | BV480 | 3/23 | BD | 746970 |
| XCR1 | BV510 | ZET | BioLegend | 148218 |
| CD11c | BV570 | N418 | BioLegend | 117331 |
| CD169 | BV605 | 3D6.112 | BioLegend | 142413 |
| F4/80 | BV650 | BM8 | BioLegend | 123149 |
| I A/E | BV711 | M5/114.15.2 | BioLegend | 107643 |
| CD117 | BV750 | 2B8 | BD | 747412 |
| CD44 | BV785 | IM7 | BioLegend | 103059 |
| CXCR5 | FITC | L138D7 | BioLegend | 145520 |
| CD8a | PerCP | 53-6.7 | BioLegend | 100732 |
| CD24 | PerCP-Cy5.5 | M1/69 | BioLegend | 101824 |
| PDCA1 | PE | REA818 | Miltenyi Biotec | 130-112-220 |
| CD103 | PE-Dazzle594 | 2E7 | BioLegend | 121430 |
| CD11b | PE-Cy5 | M1/70 | BioLegend | 101210 |
| Siglec-F | PE-Vio770 | REA798 | Miltenyi Biotec | 130-112-176 |
| CD64 | AF647 | X54-5/7.1 | BioLegend | 139322 |
| Ly6G | AF700 | 1A8 | BioLegend | 127610 |
| NK1.1 | APC-Cy7 | PK136 | BioLegend | 108724 |
| CD45 | APC-Fire810 | 30-F11 | BioLegend | 103174 |

**Supplementary Table 2**. List of antibodies used for Cytex flow cytometry-based analysis of the T cell phenotypes

| Antigen | Fluorochrome | Clone | Manufacturer | Catalog number |
|---|---|---|---|---|
| CD3 | BUV395 | 145-2C11 | BD | 563565 |
| CD69 | BV421 | H1.2F3 | BioLegend | 104528 |
| CD44 | BV510 | IM7 | BioLegend | 103044 |
| CD62L | BV570 | MEL-14 | BioLegend | 104433 |
| PD-1 | BV605 | 29F.1A2 | BioLegend | 135219 |
| ICOS | BV650 | 398.4A | BioLegend | 313550 |
| CD8 | PerCP-Cy5.5 | 53-6.7 | BioLegend | 100734 |
| CXCR5 | PE | L138D7 | BioLegend | 145504 |
| CD4 | PE-Dazzle 594 | GK1.5 | BioLegend | 100456 |
| CCR7 | PE-Cy5 | 4B12 | BioLegend | 120114 |
| CD45 | APC-Fire810 | 30-F11 | BioLegend | 103174 |

**Supplementary Table 3**. List of antibodies used for Cytex flow cytometry-based analysis of the T cell cytokine staining

| Antigen | Fluorochrome | Clone | Manufacturer | Catalog number |
|---|---|---|---|---|
| T cell surface staining | | | | |
| CD3 | BUV395 | 145-2C11 | BD | 563565 |
| CD69 | BV421 | H1.2F3 | BioLegend | 104528 |
| CD44 | BV510 | IM7 | BioLegend | 103044 |
| CD62L | BV570 | MEL-14 | BioLegend | 104433 |
| ICOS | BV650 | 398.4A | BioLegend | 313550 |
| CXCR3 | BV711 | CXCR3-173 | BD | 740825 |
| CD8 | PerCP-Cy5.5 | 53-6.7 | BioLegend | 100734 |
| CXCR5 | PE | L138D7 | BioLegend | 145504 |
| CD4 | PE-Dazzle 594 | GK1.5 | BioLegend | 100456 |
| CCR7 | PE-Cy5 | 4B12 | BioLegend | 120114 |
| PD-1 | APC | 29F.1A2 | BioLegend | 135210 |
| CD45 | APC-Fire810 | 30-F11 | BioLegend | 103174 |
| T cell intracellular cytokine staining | | | | |
| IFN-γ | BV605 | XMG1.2 | BioLegend | 505839 |
| TNF-α | BV785 | MP6-XT22 | BioLegend | 506341 |
| IL-2 | FITC | JES6-5H4 | BioLegend | 503806 |
| GrB | PE-Cy7 | QA16A02 | BioLegend | 372214 |
| IL-17A | AF700 | TC11-18H10.1 | BioLegend | 506914 |
| IL-10 | APC-Cy7 | JES5-16E3 | BioLegend | 505036 |

**Supplementary Table 4.** List of antibodies used for intracellular cytokine staining by BD LSRII flow cytometry-based analysis of the cytokine response in the splenocytes

| Antigen | Fluorochrome | Clone | Manufacturer | Catalog number |
|---|---|---|---|---|
| CD3 | BUV737 | 145-2C11 | BD | 612771 |
| CD4 | BUV395 | GK1.5 | BD | 565974 |
| CD8a | BV711 | 53-6.7 | BD | 563046 |
| IFN-γ | V450 | XMG1.2 | BD | 560661 |
| IL-4 | PerCP-Cy5 | 11B11 | BD | 560700 |
| TNF-α | FITC | MP6-XT22 | BD | 554418 |
| IL-2 | APC | JES6-5H4 | Biolegend | 503810 |
| IL-2 | PE | JES6-5H4 | Biolegend | 503808 |

**Supplementary Table 5.** List of antibodies used for staining perfused lung cells and analysis by BD LSRII flow cytometry

| Antigen | Fluorochrome | Clone | Manufacturer | Catalog number |
|---|---|---|---|---|
| CD3 | BUV737 | 145-2C11 | BD | 612771 |
| CD4 | BUV395 | GK1.5 | BD | 565974 |
| CD8a | BV711 | 53-6.7 | BD | 563046 |
| CD69 | BV650 | H1.2F3 | BD | 740460 |
| CD103 | BV510 | 2E7 | BD | 748258 |

**Supplementary Table 6**. List of MHC class I tetramer used for the detection of SARS-CoV-2 spike specific $CD8^+$ T cell response

| Allele | Description[1] | Peptide sequence | Fluorochrome | Concentration |
|---|---|---|---|---|
| H-2K$^d$ | SARS-CoV-2-spike aa 365-373 | CYGVSPTKL | PE-labeled tetramer | 1.1 mg/mL |
| H-2K$^b$ | SARS-CoV-2-spike aa 433-441 | GNYNYLYRL | PE-labeled tetramer | 1.3 mg/mL |
| H-2K$^b$ | SARS-CoV-2-spike aa 539-546 | VNFNFNGL | PE-labeled tetramer | 1.2 mg/mL |
| H-2K$^b$ | SARS-CoV-2-spike aa 435-443 | YNYLYRLF | PE-labeled tetramer | 1.3 mg/mL |

[1]The tetramers were obtained from NIH Tetramer Core Facility at Emory University, Atlanta, GA.
