# Supplementary material for: SARS-CoV-2 ferritin nanoparticle vaccine induces robust innate immune activity driving polyfunctional spike-specific T cell responses
Source: NPJ Vaccines. 2021 Dec 13;6:151. doi: 10.1038/s41541-021-00414-4 (PMC8668928; doi:10.1038/s41541-021-00414-4)
Supplement: Supplementary file 2 — Reporting Summary [file 41541_2021_414_MOESM2_ESM.pdf]

nature portfolio

Corresponding author(s): Rao, Mangala and Malloy, Allison

Last updated by author(s): Oct 8, 2021

Reporting Summary

Nature Portfolio wishes to improve the reproducibility of the work that we publish. This form provides structure for consistency and transparency in reporting. For further information on Nature Portfolio policies, see our Editorial Policies and the Editorial Policy Checklist.

Statistics

For all statistical analyses, confirm that the following items are present in the figure legend, table legend, main text, or Methods section.

n/a	Confirmed	
☐	☒	The exact sample size (n) for each experimental group/condition, given as a discrete number and unit of measurement
☐	☒	A statement on whether measurements were taken from distinct samples or whether the same sample was measured repeatedly
☐	☒	The statistical test(s) used AND whether they are one- or two-sided<br>*Only common tests should be described solely by name; describe more complex techniques in the Methods section.*
☒	☐	A description of all covariates tested
☒	☐	A description of any assumptions or corrections, such as tests of normality and adjustment for multiple comparisons
☐	☒	A full description of the statistical parameters including central tendency (e.g. means) or other basic estimates (e.g. regression coefficient) AND variation (e.g. standard deviation) or associated estimates of uncertainty (e.g. confidence intervals)
☐	☒	For null hypothesis testing, the test statistic (e.g. F, t, r) with confidence intervals, effect sizes, degrees of freedom and P value noted<br>*Give P values as exact values whenever suitable.*
☒	☐	For Bayesian analysis, information on the choice of priors and Markov chain Monte Carlo settings
☒	☐	For hierarchical and complex designs, identification of the appropriate level for tests and full reporting of outcomes
☐	☒	Estimates of effect sizes (e.g. Cohen's d, Pearson's r), indicating how they were calculated

Our web collection on statistics for biologists contains articles on many of the points above.

Software and code

Policy information about availability of computer code

Data collection	Flow cytometry data was collected on a 5-laser Cytek Aurora flow cytometer (Cytek Biosciences) and BD LSR II (BD Biosciences).<br>ELIspot plates were counted and data analyzed using the AID Autoimmun Diagnostica GmbH ELISpot reader.<br>Cytokine levels were measured using MSD V-Plex Plus Multi-Spot Assay plates, read by MESO SECTOR S 120 Reader from Meso Scale Discovery (MSD, Rockville, MD)
Data analysis	Flow cytometry data was accquired by SpectroFlo® software and BD FACS DIVA software, and analyzed using FlowJo software v10 (Tree Star, Inc.).<br>MSD based cytokine or analyte concentration was calculated using DISCOVERY WORKBENCH®MSD Software.<br>Statistical analyses were conducted using GraphPad Prism v.8.4.0 software.

For manuscripts utilizing custom algorithms or software that are central to the research but not yet described in published literature, software must be made available to editors and reviewers. We strongly encourage code deposition in a community repository (e.g. GitHub). See the Nature Portfolio guidelines for submitting code & software for further information.

Data

Policy information about availability of data

All manuscripts must include a data availability statement. This statement should provide the following information, where applicable:

- Accession codes, unique identifiers, or web links for publicly available datasets
- A description of any restrictions on data availability
- For clinical datasets or third party data, please ensure that the statement adheres to our policy

Data generated or analyzed during this study are included in this article and its supplementary information files.

Field-specific reporting

Please select the one below that is the best fit for your research. If you are not sure, read the appropriate sections before making your selection.

☒ Life sciences ☐ Behavioural & social sciences ☐ Ecological, evolutionary & environmental sciences

For a reference copy of the document with all sections, see nature.com/documents/nr-reporting-summary-flat.pdf

Life sciences study design

All studies must disclose on these points even when the disclosure is negative.

Sample size	In order to assure statistical significance in the results at-least N=5 mice/ group /time point were taken. Each experiment was repeated at-least twice independently. These have been described clearly in the methods section. For flow cytometry experiments samples from individual mice (n=5 or 10) were analyzed and two independent experiments were performed.
Data exclusions	No data were excluded.
Replication	Time points days 3, 5, and 10 were repeated twice, each time with an n=5 mice/time point/adjuvant. The 6-week time point with SpFN+ALFQ vaccine was repeated twice (n=5, n=9).<br>Each sample was run in triplicates for Elispot and MSD assays.
Randomization	All animals were randomly assigned to vaccination groups.
Blinding	Blinding was not performed, however the experiments were conducted in an unbiased manner to prevent potential biases in the experimental groups.

Reporting for specific materials, systems and methods

We require information from authors about some types of materials, experimental systems and methods used in many studies. Here, indicate whether each material, system or method listed is relevant to your study. If you are not sure if a list item applies to your research, read the appropriate section before selecting a response.

Materials & experimental systems

n/a	Involved in the study	
☐	☒	Antibodies
☐	☒	Eukaryotic cell lines
☒	☐	Palaeontology and archaeology
☐	☒	Animals and other organisms
☒	☐	Human research participants
☒	☐	Clinical data
☒	☐	Dual use research of concern

Methods

n/a	Involved in the study	
☒	☐	ChIP-seq
☐	☒	Flow cytometry
☒	☐	MRI-based neuroimaging

Antibodies

Antibodies used

Antigen Fluorochrome Clone Manufacturer Catalog number
CD45R/B220 BUV395 RA3-6B2 BD 563793
CD80 BUV661 16-10A1 BD 741515
CD3 BUV737 145-2C11 BD 612771
CD86 BUV805 GL1 BD 741946
CCR7 BV421 4B12 BioLegend 120120
CD40 BV480 3/23 BD 746970

XCR1 BV510 ZET BioLegend 148218
CD11c BV570 N418 BioLegend 117331
CD169 BV605 3D6.112 BioLegend 142413
F4/80 BV650 BM8 BioLegend 123149
I A/E BV711 M5/114.15.2 BioLegend 107643
CD117 BV750 2B8 BD 747412
CD44 BV785 IM7 BioLegend 103059
CXCR5 FITC L138D7 BioLegend 145520
CD8a PerCP 53-6.7 BioLegend 100732
CD24 PerCP-Cy5.5 M1/69 BioLegend 101824
PDCA1 PE REA818 Miltenyi Biotec 130-112-220
CD103 PE-Dazzle594 2E7 BioLegend 121430
CD11b PE-Cy5 M1/70 BioLegend 101210
Siglec-F PE-Vio770 REA798 Miltenyi Biotec 130-112-176
CD64 AF647 X54-5/7.1 BioLegend 139322
Ly6G AF700 1A8 BioLegend 127610
NK1.1 APC-Cy7 PK136 BioLegend 108724
CD45 APC-Fire810 30-F11 BioLegend 103174
CD3 BUV395 145-2C11 BD 563565
CD69 BV421 H1.2F3 BioLegend 104528
CD44 BV510 IM7 BioLegend 103044
CD62L BV570 MEL-14 BioLegend 104433
PD-1 BV605 29F.1A2 BioLegend 135219
ICOS BV650 398.4A BioLegend 313550
CD8 PerCP-Cy5.5 53-6.7 BioLegend 100734
CXCR5 PE L138D7 BioLegend 145504
CD4 PE-Dazzle 594 GK1.5 BioLegend 100456
CCR7 PE-Cy5 4B12 BioLegend 120114
CD45 APC-Fire810 30-F11 BioLegend 103174
T cell surface staining
CD3 BUV395 145-2C11 BD 563565
CD69 BV421 H1.2F3 BioLegend 104528
CD44 BV510 IM7 BioLegend 103044
CD62L BV570 MEL-14 BioLegend 104433
ICOS BV650 398.4A BioLegend 313550
CXCR3 BV711 CXCR3-173 BD 740825
CD8 PerCP-Cy5.5 53-6.7 BioLegend 100734
CXCR5 PE L138D7 BioLegend 145504
CD4 PE-Dazzle 594 GK1.5 BioLegend 100456
CCR7 PE-Cy5 4B12 BioLegend 120114
PD-1 APC 29F.1A2 BioLegend 135210
CD45 APC-Fire810 30-F11 BioLegend 103174
T cell intracellular cytokine staining
IFN-⍰ BV605 XMG1.2 BioLegend 505839
TNF-⍰ BV785 MP6-XT22 BioLegend 506341
IL-2 FITC JES6-5H4 BioLegend 503806
GrB PE-Cy7 QA16A02 BioLegend 372214
IL-17A AF700 TC11-18H10.1 BioLegend 506914
IL-10 APC-Cy7 JES5-16E3 BioLegend 505036
CD3 BUV737 145-2C11 BD 612771
CD4 BUV395 GK1.5 BD 565974
CD8a BV711 53-6.7 BD 563046
IFN-⍰ V450 XMG1.2 BD 560661
IL-4 PerCP-Cy5 11B11 BD 560700
TNF-⍰ FITC MP6-XT22 BD 554418
IL-2 APC JES6-5H4 Biolegend 503810
IL-2 PE JES6-5H4 Biolegend 503808
CD3 BUV737 145-2C11 BD 612771
CD4 BUV395 GK1.5 BD 565974
CD8a BV711 53-6.7 BD 563046
CD69 BV650 H1.2F3 BD 740460
CD103 BV510 2E7 BD 748258

Validation

All antibodies are validated by their respective manufacturer and were titrated prior to use.

Eukaryotic cell lines

Policy information about cell lines

Cell line source(s)

mammalian Expi293 cells (Thermo Fisher Scientific).

Authentication

Commercially purchased (Thermo Fisher Scientific) and previously used in several experiments.

Mycoplasma contamination	Negative for mycoplasma.
Commonly misidentified lines (See ICLAC register)	None were utilized.

Animals and other organisms

Policy information about studies involving animals; ARRIVE guidelines recommended for reporting animal research

Laboratory animals	Female C57BL/6 mice (5-6 weeks of age) were obtained from The Jackson Laboratory.
Wild animals	No wild animals were included.
Field-collected samples	No field collected animals were included.
Ethics oversight	Animal studies were carried out in accordance with the recommendations in the Guide for the Care and Use of Laboratory Animals of the National Institutes of Health.<br>The protocols were approved by the Institutional Animal Care and Use Committee at the Walter Reed Army Institute of Research [Assurance number D16-00596 (A4117-01)]. All this information is provided in the method section of the manuscript.

Note that full information on the approval of the study protocol must also be provided in the manuscript.

Flow Cytometry

Plots

Confirm that:

- [ ] The axis labels state the marker and fluorochrome used (e.g. CD4-FITC).
- [x] The axis scales are clearly visible. Include numbers along axes only for bottom left plot of group (a 'group' is an analysis of identical markers).
- [x] All plots are contour plots with outliers or pseudocolor plots.
- [x] A numerical value for number of cells or percentage (with statistics) is provided.

Methodology

Sample preparation	Cryopreserved or fresh splenocytes and lymph nodes cells were used for the flow cytometry experiments described clearly in the method section.
Instrument	Cytek Aurora flow cytometer (Cytek Biosciences) and BD LSR II (BD Biosciences).
Software	SpectroFlo® software<br>BD FACS DIVA software<br>FlowJo software v10 (Tree Star, Inc.).
Cell population abundance	No sorting was performed.
Gating strategy	Gating strategies are provided in Supplementary figures.

- [x] Tick this box to confirm that a figure exemplifying the gating strategy is provided in the Supplementary Information.
